# Determinants of de novo mutations in extended pedigrees of 43 dog breeds

**DOI:** 10.1186/s13059-025-03804-2

**Published:** 2025-09-25

**Authors:** Shao-Jie Zhang, Jilong Ma, Meritxell Riera, Søren Besenbacher, Julia E. Niskanen, Noora Salokorpi, Sruthi Hundi, Marjo K. Hytönen, Tong Zhou, Gui-Mei Li, Elaine A. Ostrander, Mikkel Heide Schierup, Hannes Lohi, Guo-Dong Wang

**Affiliations:** 1 State Key Laboratory of Genetic Evolution & Animal Models, Kunming Institute of Zoology, Chinese Academy of Sciences, Kunming, China; 2 Kunming College of Life Science, University of Chinese Academy of Sciences, Kunming, China; 3 Bioinformatics Research Centre, Aarhus University, Aarhus C, 8000 Denmark; 4 Department of Biology, Aarhus University, Aarhus C, 8000 Denmark; 5 Department of Molecular Medicine, Aarhus University Hospital, Aarhus N, 8200 Denmark; 6 Department of Clinical Medicine, Aarhus University, Aarhus C, 8000 Denmark; 7 Department of Medical and Clinical Genetics, Faculty of Medicine, University of Helsinki, Helsinki, Finland; 8 Department of Veterinary Biosciences, Faculty of Veterinary Medicine, University of Helsinki, Helsinki, Finland; 9 Folkhälsan Research Center, Helsinki, Finland; 10 National Human Genome Research Institute, National Institutes of Health, Bethesda, MD 20892 USA; 11 Yunnan Key Laboratory of Molecular Biology of Domestic Animals, Kunming, China

**Keywords:** De novo mutation, Dog breeds, Mutation rate, *PRDM9*, CpG Islands, *MLH1*

## Abstract

**Background:**

Understanding the determinants of de novo mutation is critical for elucidating evolutionary processes and genetic disease susceptibility. But the interplay between life history, genomic architecture, and recombination remains poorly understood in non-model species. Domestic dogs, lacking the recombination regulator *PRDM9* and subject to intense artificial selection, offer a unique system for dissecting factors that jointly influence mutation accumulation. Here, we leverage large-scale trio sequencing to unravel the determinants of de novo mutations across diverse dog breeds.

**Results:**

By analyzing 390 trios from 43 breeds, we estimate a germline mutation rate of 4.89 × 10^−9^ per base pair per generation. Parental age, especially paternal age, strongly influences mutation rates, with a 1.5-fold greater paternal age effect in dogs compared to humans. Larger breeds exhibit elevated early-life mutations, aligning with accelerated developmental trajectories. Strikingly, CpG Islands in dogs exhibit a 2.6-fold higher mutation rate than the genomic average, unlike humans where no such increase occurs. We also find a tenfold hypermutated dog and suggest a unique maternal mechanism of *MLH1*-mediated germline instability during gametogenesis.

**Conclusions:**

The unique mutational landscape in canids is determined by paternal age, body size, and CpG Islands recombination. Despite extensive breeding, germline mutation rates in dogs remain stable across breeds. The elevated mutation rate in CpG Islands due to recombination in the absence of *PRDM9* underscores a distinct evolutionary mechanism in canids. These findings enhance our understanding of mutation dynamics, with implications for canine genetic diversity, disease susceptibility, and broader genomic studies in species lacking *PRDM9*.

**Supplementary Information:**

The online version contains supplementary material available at 10.1186/s13059-025-03804-2.

## Background

Decreased costs of genome sequencing and improved bioinformatics pipelines [1] have made it possible to directly identify de novo mutations by sequencing parent–offspring trios at scale. This has led to mutation rate estimates for many vertebrates [2–5], improving phylogenetic dating and providing insights into evolutionary changes to the mutational process across vertebrates. While some species have been studied for mutation rates using a limited number of trios [2–5], only humans [6, 7] and mice [8] have been studied with sufficient numbers of trios sequenced and analyzed to allow for intraspecific investigation of factors influencing mutation rates. Consequently, little is known about how mutations accumulate over time in the germline of other mammalian species, such as domestic dogs (*Canis lupus familiaris*).

Canids constitute a fascinating system for the study of mutational processes. They are unique among mammals in that they lack a functional *PRDM9* ortholog [9]. In other mammals, *PRDM9* recognizes specific sequence motifs and directs the recombination machinery toward these positions, though in some species only weakly [10]. In canids, without *PRDM9*, recombination is directed to open chromatin regions, most notably CpG Islands (CGIs) [9, 11]. Since recombination is mutagenic [6], the lack of *PRDM9*-directed recombination in dogs should translate into differences in the distribution of germline de novo mutations (DNMs) compared to other mammalian species with a functional *PRDM9* gene, such as humans.

Intensive breeding of dogs in the past 200 years has fostered an impressive diversity in morphologic features, e.g., body size [12, 13], shape [14], fur type [15, 16], coat color [17, 18], and skull shape [19], as well as breed-enriched behaviors and disease susceptibility [14, 20–22]. Association studies across breeds have identified alleles of considerable impact compared to the frequently observed minor impact variants identified in comparative human studies [12]. Whether intensive artificial selection has also affected the mutation rate in individual breeds is not known.

Here, we identify de novo mutations in 390 trios from 43 breeds of dogs raised in similar environments in Finland. Our dataset includes large pedigrees (on average 7.48 trios), allowing us to study potential differences in mutational processes among breeds with different phenotypical makeups and life histories. Moreover, by comparing the accumulation of germline DNMs in CGIs with the rest of the genome, we can estimate the mutagenic effects of recombination in dogs and predict when *PRDM9*-directed recombination was lost in the canid lineage.

## Results

### Dog mutation rates shaped by parental ages

**Fig. 1:**
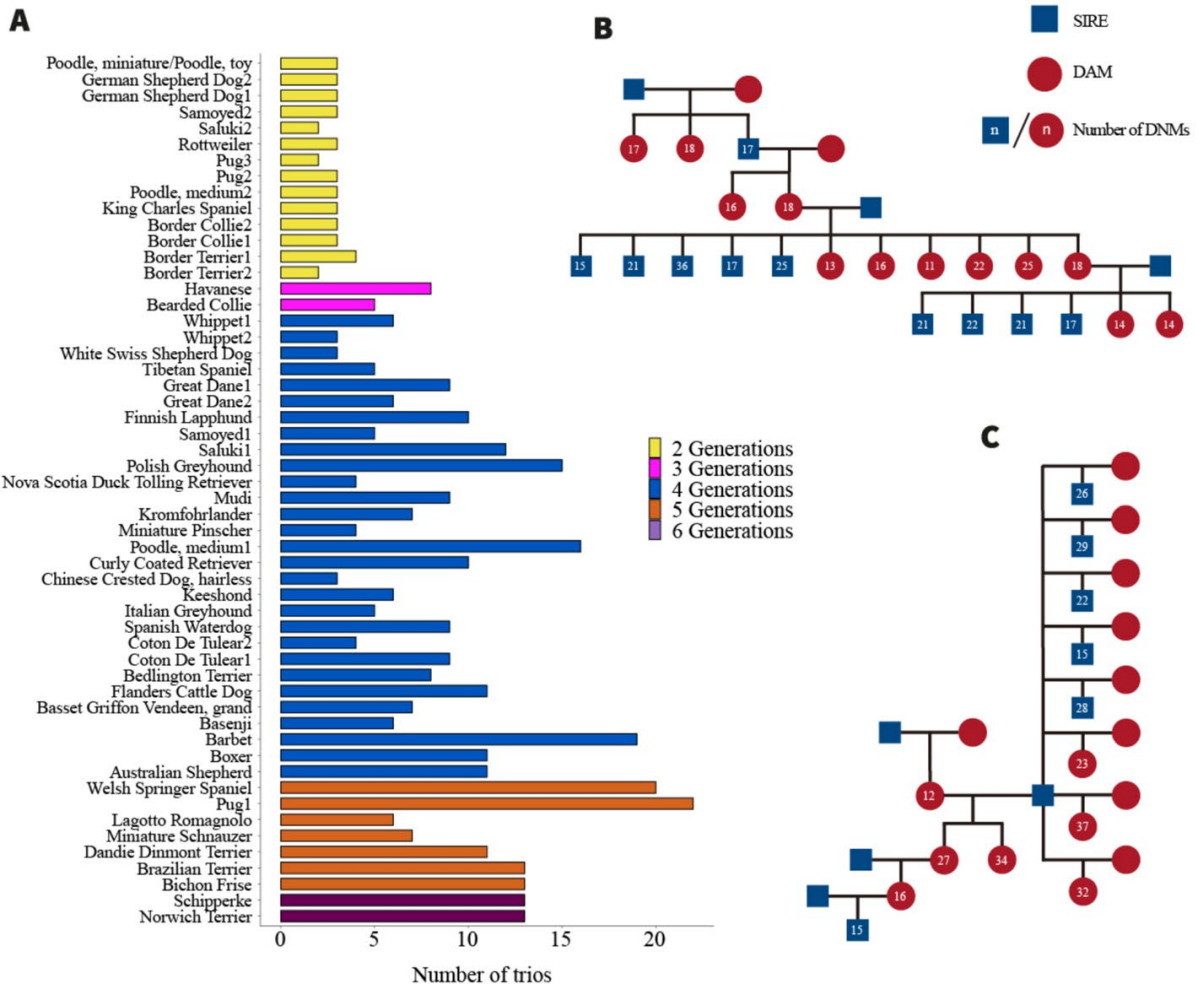
Sample information and statistics of de novo mutations. **A** Number of trios per pedigree. Bar graphs show the corresponding breeds for all 54 pedigrees (horizontal axis), the number of trios in each pedigree (vertical axis), and the number of generations in each pedigree (color of bars). **B** and **C** Two representative pedigrees: pug1 and Bichon Frisé. Blue boxes represent males, red circles represent females, and the numbers in circles represent the number of DNMs for the individual

We sequenced dog families collected at the dog biobank at the University of Helsinki, Finland, over the past ~ 15 years. Genomes were sequenced to an average coverage of 43.3 × from 643 dogs (341 females, 302 males) representing 54 multigenerational families and 404 trios, from 43 distinct breeds (Additional file 1: Note S1, Additional file 2: Data S1). We excluded 14 trios with at least one individual displaying an average sequence coverage lower than 24 ×, thus retaining 390 trios. The pedigrees vary in relationship structure and size, including 37 extended pedigrees, with an average litter size of 2.4, and 81 trios with multiple siblings, with an average litter size of 3.6 (Fig. 1A and B). We applied a stringent pipeline to call DNMs for all 404 trios (Methods and Additional file 1: Fig. S1), identifying 8312 high-quality autosome DNMs (Additional file 2: Data S2) in 390 trios, with an average of 21.31 DNMs per trio (95% CI: 20.14–22.49, Binomial), and a mean callable genome fraction of 96.52% (95% CI 96.44–96.56, Bootstrap).

We found that 1.51% of DNMs are in coding regions, similar to what has been previously reported in humans (Additional file 1: Fig. S2). Searching for genes with several mutations, we found enrichment for neurodevelopmental genes in both dogs and humans, but these were generally in non-coding regions, requiring further investigation to determine if these regions play key regulatory roles and how the observed variants affect function (Additional file 1: Note S2, Additional file 2: Data S3–S6).

**Fig. 2:**
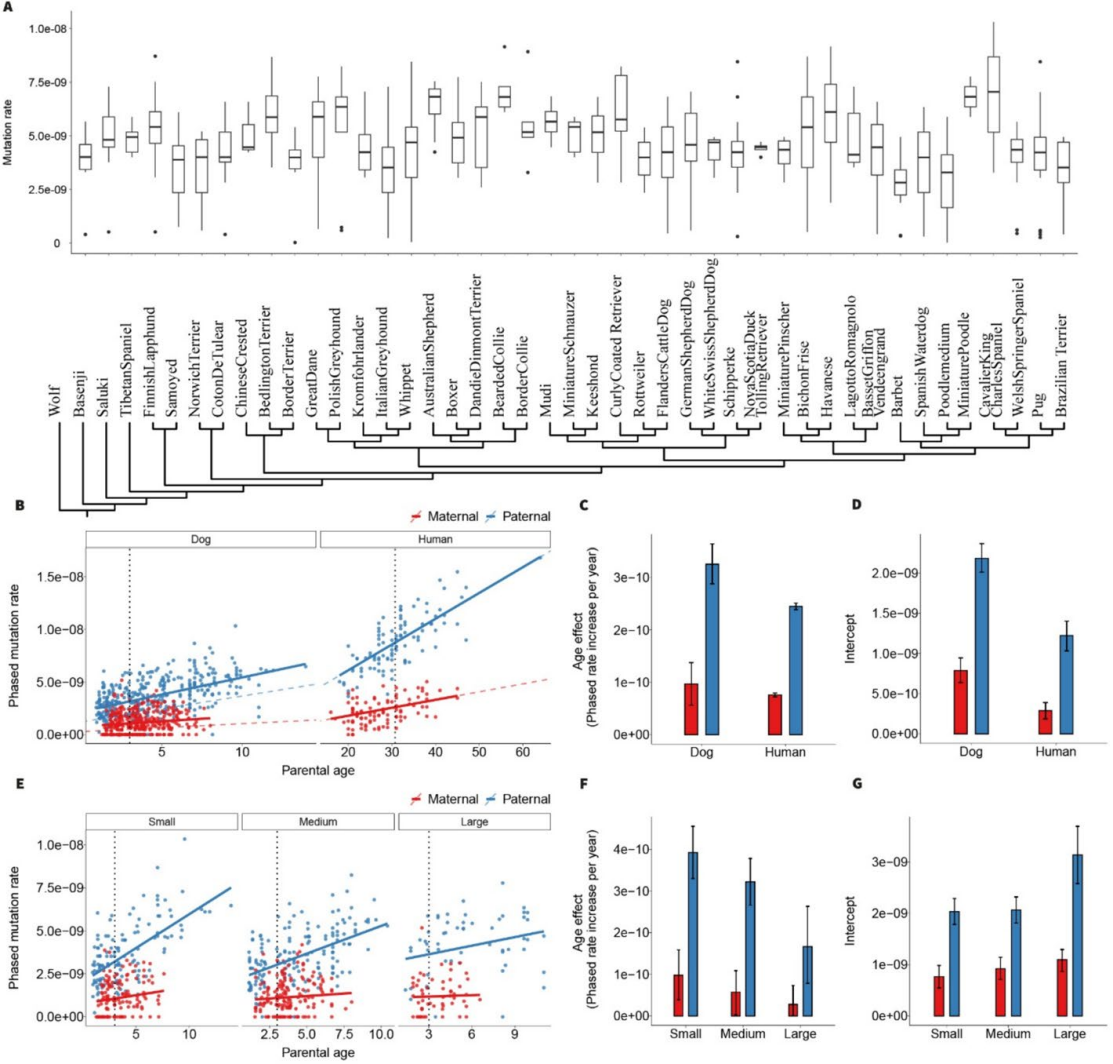
Demonstrations of mutation rates. **A** Mutation rates from individual breeds and their phylogenetic relationships. **B** Phased mutation rates with parental age among humans and dogs. **C** The age effect of the mutation rate for paternal and maternal in dogs and humans, respectively. **D** Intercept of the mutation rate for paternal and maternal in dogs and humans, respectively. **E** Phased mutation rates with parental age among small, intermediate, and large dog breeds. **F** The age effect of the mutation rate for paternal and maternal among small, intermediate, and large dog breeds. **G** Intercept of the mutation rate for paternal and maternal among small, intermediate, and large dog breeds

Focusing on individual callable genome (Methods), we observe an average germline DNM rate of 4.89 × 10^−9^ (95% CI 4.77 × 10^−9^–5.02 × 10^−9^, Bootstrap) per base pair, per generation, across autosomes. This estimate is close to previous pedigree-based estimates (4.5 × 10^−9^), which are based on de novo mutation analyses in a pedigree of seven wolves [23]. Recalibrating the demographic history model of dogs and wolves using our new mutation rate, we now estimate the divergence time to be 23,000–30,000 years. Because our mutation rate estimate is more precise (lower bound 4.77 × 10^−9^, upper bound 5.02 × 10^−9^), the credible interval for the dog–wolf divergence shrinks from the original Koch et al. estimate of 16–64 ka [23] to approximately 14–40 ka (Table S1). Figure 2A demonstrates per-generation mutation rate estimates from individual breeds, together with their phylogenetic relationships. The estimated mutation rate per trio differs significantly among breeds (*P* = 5.4 × 10^−12^, ANOVA). The breed’s effect on the rate per trio is reduced after accounting for differences in paternal age at conception but remains statistically significant (*P* = 0.00014, ANOVA). However, those differences are no longer significant after accounting for rates per litter, instead of treating littermates as independent trios (*P* = 0.602, ANOVA) (Additional file 1: Note S3).

Using read-backed phasing, we determined the parental origins of 2586 out of 8312 DNMs (31.11%). Of the phased DNMs, 75.05% (95% CI 73.59–76.48, Bootstrap) are of paternal origin, corresponding to a male-to-female mutation ratio of 3.01 (95% CI 2.79–3.25, Bootstrap), compared to 79% paternal origin (ratio 3.70) reported in humans [7]. We investigated paternal and maternal DNMs as a function of parental ages (Additional file 1: Fig. S3) and compared the dog results to human data [6]. We find that paternal age and maternal age are both significant predictors of mutation rate in dogs (Additional file 1: Fig. S4) (adjusted *R*^2^ = 0.3425, *P* < 2 × 10^−16^ and *P* = 0.000118, respectively), as in humans. We also modeled phased mutation rates as a function of parental ages using Bayesian Poisson regression (Fig. 2B). We observe significant posterior estimates for paternal age effects on paternal mutation rates (3.25 × 10^−10^, 95% HDI 2.88 × 10^−10^–3.63 × 10^−10^) and maternal age effects on maternal mutation rates (9.64 × 10^−11^, 95% HDI 5.61 × 10^−11^–1.37 × 10^−10^) (Additional file 1: Note S4). Dogs show a steeper accumulation of paternal mutations per year, with paternal age effect estimates 1.5 times greater than in humans (Fig. 2C). This higher yearly accumulation in the male germline and their shorter generation time translates into a 3.7 times higher yearly rate of de novo mutations in dogs compared to humans (1.41 × 10^−9^ HDI: 1.37 × 10^−9^–1.45 × 10^−9^ vs 3.8 × 10^−10^ HDI: 3.78 × 10^−10^–3.82 × 10^−10^), comparing to the rate from phylogenetic comparisons in dogs (3.33 × 10^−9^) [24] is 3.33 times than that in humans (1.0 × 10^−9^) [7]. Additionally, the posterior estimates for paternal intercepts are higher in dogs than in humans (Fig. 2D). While the intercepts involve contributions from early-acquired mutations, we notice that the intercept estimations have no power in quantifying early mutation metrics as they are highly confounded by the age effect estimation in the regression model. Predictions of paternal mutation rate at puberty are approximately twofold higher in humans than in dogs, despite humans having a more than tenfold higher puberty age than dogs (Additional file 1: Note S5).

Paternal age at conception explains less of the variance in paternal germline mutation rates in dogs (McFadden’s *R*^2^ of 30.47%) than in humans (McFadden’s *R*^2^ of 56.18%), as calculated using Poisson regression. McFadden’s *R*^2^ value is still higher in humans after downsampling the number of DNMs to match that found in dogs (56.06% vs 30.47%, Additional file 1: Note S4), suggesting that additional factors contribute to the variance in the accumulation of paternal DNMs in dogs. We tested for differences in 21 quantitative phenotypes among breeds, including size and lifespan, but found none with a significant effect on the overall mutation rate per litter, based on an ANOVA analysis using parental ages as covariates. (Additional file 1: Note S3).

Next, we examined the relationship between parental age and the accumulation of germline DNMs across dog breeds of different sizes (Fig. 2E), categorizing each breed as small, intermediate, or large based on average adult weight (based on Fédération Cynologique Internationale standard). The model estimates a stronger paternal age effect in small breeds, with a higher rate of mutation accumulation per year (3.93 × 10^−10^, HDI: 3.30 × 10^−10^–4.55 × 10^−10^) than in large breeds (1.66 × 10^−10^, HDI: 7.81 × 10^−11^–2.63 × 10^−10^) (Fig. 2F). Consequently, the model estimates a higher intercept for large breeds (3.14 × 10^−9^, HDI: 2.58 × 10^−9^–3.7 × 10^−9^) than for small breeds (2.03 × 10^−9^, HDI: 1.78 × 10^−9^–2.29 × 10^−9^) (Fig. 2G), while the paternal mutation rate predicted at puberty age reveals no significant difference among dog breeds of different sizes (Additional file 1: Note S5).These findings imply that although the per-generation mutation rate is broadly similar across breed sizes, the dynamics of germline DNM accumulation over a dog’s lifetime may differ with body size. Nevertheless, despite the model yielding distinct intercepts and age-effect estimates for different size categories, leave-one-out cross-validation reveals no significant difference in predictive accuracy between models with shared versus size-specific parameters (Additional file 1: Table 2, Additional file 1: Note S5).

### Putative maternal *MLH1*-mediated hypermutation

Our dataset includes a hypermutated individual (ID: FAM007647) with 214 DNMs, showing a strong maternal bias: 140 maternally derived, 13 paternally derived, and 61 unphased. This was the only hypermutated case observed across the cohort. While the number of DNMs per individual in other trios ranged from 21 to 25, this individual exhibited nearly a tenfold excess. The trio was sequenced at 50 × coverage, and all samples passed stringent quality control and filtering to exclude technical artifacts. Parentage was confirmed using Mendelian inheritance of DNMs, ruling out contamination or sample mix-up. If there were contamination from other samples, we would see a significantly higher number of DNMs.

The proband and both parents are purebred Border Collies with complete pedigrees. The hypermutated dog was bred in Finland. The sire, imported from the UK, belongs to a long-established British Border Collie lineage. The dam was born in Finland, with both parents imported from well-known Australian show lines. The hypermutated dog died at 14 years of age with pancreatitis, and cancer was also suspected. The sire died at 14, and the dam at 13; neither had any notable health issues during their lifetimes. No unusual environmental exposures were reported that could account for the excess DNMs, suggesting a potential endogenous cause.

Germline variant calling from raw FASTQ files revealed that the sire and proband had typical levels of germline variation relative to the entire pedigree cohort, but the dam had approximately 30% more germline SNVs. While its transition/transversion (Ti/Tv) ratio was 2.05, all other Border Collies in the pedigree cohort had Ti/Tv ratios ranging between 2.01 and 2.04. Previous canine whole genome studies [12, 25] suggest that a Ti/Tv ratio ~ 2.0 represents true biological variants rather than artifacts; therefore, the Ti/Tv ratio of 2.05 is also within the normal range.

Recent human studies demonstrate that hypermutation can arise from defects in DNA repair genes or parental exposure to mutagenic agents. Kaplanis et al. analyzed nearly 22,000 human trios and identified individuals with a 2–7 × increase in DNMs [26]; in several instances, damaging variants in DNA repair genes or paternal chemotherapy were implicated, providing a clear rationale for exploring DNA repair mechanisms in our canine cohort. We screened the trio and three other Border Collies for exonic and loss-of-function variants in genes implicated in DNA repair, including those associated with base excision repair (*MBD4*,* MUTYH*,* OGG1*,* TDG*,* NTHL1*), proofreading polymerases (*POLE*,* POLD1*), mismatch repair (*MLH1*,* PMS2*,* MSH2*,* MSH6*), oxidative damage response (*NEIL1–3*,* APEX1*), double-strand break repair (*BRCA1*,* BRCA2*,* CHEK2*,* NBN*), and nucleotide excision repair (*XPC*,* ERCC2*,* ERCC5*). No relevant variants were found in the sire, offspring, and three other Border Collies. However, the dam harbored a heterozygous missense variant (p.D375N) in *MLH1*, predicted to be probably damaging by PolyPhen-2 [27] and classified as pathogenic by AlphaMissense [28] based on the aligned human residue. This variant was not transmitted to the offspring and was absent in other Border Collies in the entire pedigree cohort.

Together, the elevated mutation load, strong maternal bias, and presence of a potentially pathogenic *MLH1* variant in the dam suggest a model of transient mismatch repair deficiency during maternal gametogenesis. The hypermutated dog was excluded from downstream analyses to avoid skewing association tests or mutation rate estimates.

### Mutational spectrum in dogs compared to humans

**Fig. 3:**
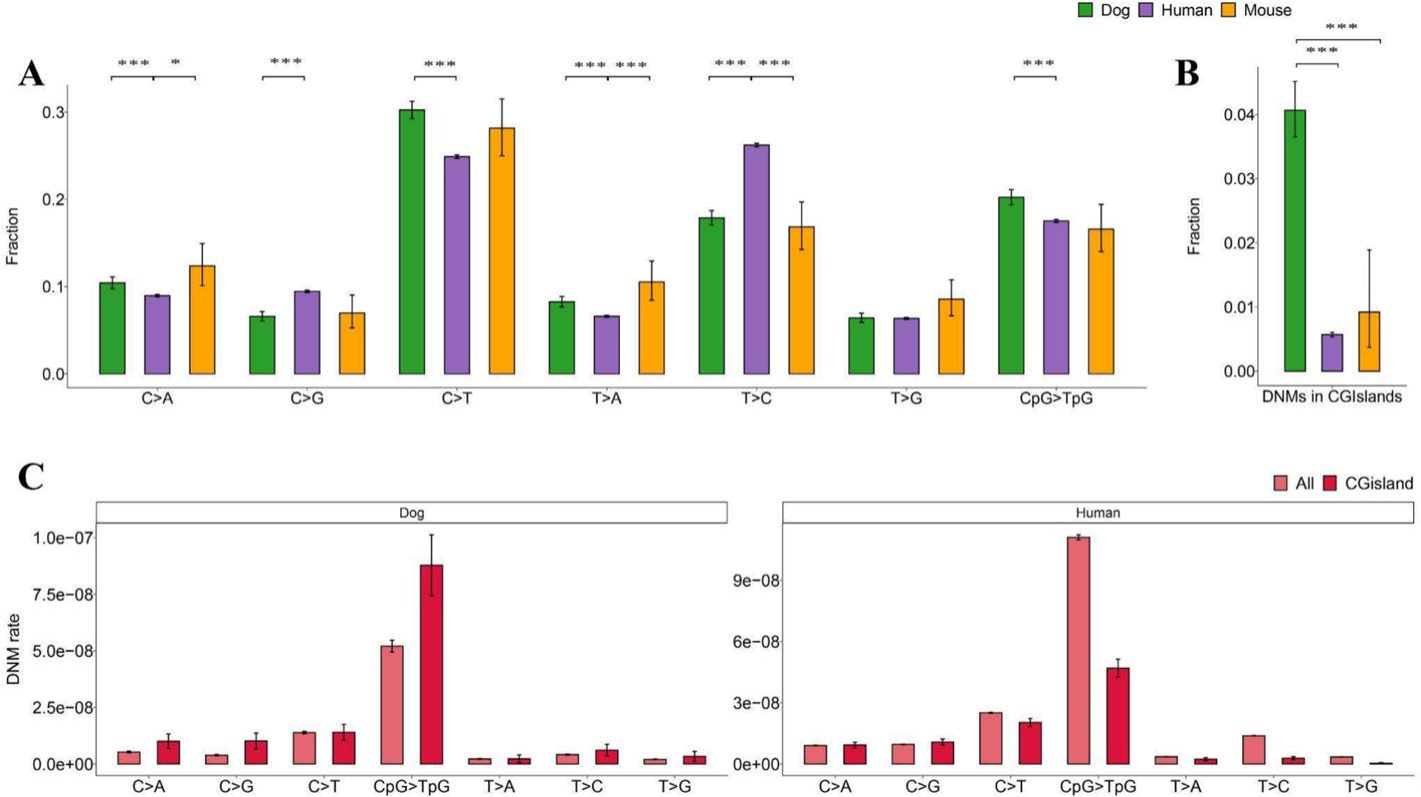
Mutational spectrum among dogs and other species. **A** Relative frequency of mutational classes by mouse, dog, and human. **B** Fractions of DNMs found in CGIs in mouse [8], dog, and human [6] trio studies. **C** Comparison of mutation rates of different mutation classes in CGIs and the autosome in humans and dogs

We next compared the mutational spectrum of germline DNMs in dogs, mice, and humans by stratifying DNMs into seven classes representing the six possible single base pair changes, plus C > T mutations in a CpG context (Methods) (Fig. 3A). The spectra of mutations show a much higher similarity between dogs and mice, despite a closer phylogenetic relationship between humans and mice (Fig. 3A, Additional file 2: Data S7). Notably, dogs show a more significant fraction of C > T mutations and a smaller fraction of T > C mutations than humans. The fraction of C > T and T > C DNMs has been shown to decrease and increase, respectively, with paternal age in humans [7]. This difference in mutation profiles has also been observed when comparing paternally transmitted mutations accumulated early in development, i.e., those shared between siblings, with DNMs accumulating later in development versus those not shared [29]. In line with these observations, we find that the fraction of C > T DNMs in dogs also decreases with increasing paternal age (*P* = 0.02, Additional file 1: Fig. S5). We hypothesize that the overall differences in the mutational spectrum observed in dogs could be explained by a higher proportion of mutations in dogs occurring early in development, i.e., before puberty, as suggested by the significantly larger intercept in the accumulation of DNMs with parental age. To further investigate this possibility, we identified DNMs shared between siblings or half-siblings. We find that the fraction of shared mutations among dog siblings (79 out of 8312) is similar to that observed in a comparable human trio data set (548 out of 47,585) [29] (Additional file 1: Fig. S6), which translates to a sibling shared mutation fraction of 0.45% (95% CI 0.34–0.58%) in dogs compared to 0.4% (95% CI 0.1–0.7%) in humans. The similarly low sibling shared mutation fraction suggests no difference in fractions of early mutations between humans and dogs. We also compared the mutational composition of DNMs shared between siblings (77) or half-siblings (2), but found no significant differences in the mutational spectrum of shared and non-shared mutations in bootstrapping (Fig. S6, Additional file 2: Data S8). However, it is worth noting that the test is underpowered since it is based on only 79 unique mutations shared by 34 sibling groups. There are 23 out of 77 full-sibling shared mutations that are phased to parental sources, revealing no paternal bias in the sibling shared mutations (11 maternal and 12 paternal, *P* = 0.48, binomial), which reconciles with the hypothesis that early developmental mutations accrue equally in both sexes [30].

Interestingly, in CpG island regions, dogs have significantly more DNMs than humans or mice (7.14 times higher than humans, *P* < 2.2 × 10^−16^; 4.41 times higher than mice, *P* = 7.949 × 10^−7^) (Fig. 3B). We also compared the mutation rate of seven mutational spectra between the CpG island regions and all autosomal regions in dogs and humans, respectively (Fig. 3C). We found that the CpG > TpG mutation rate in CGIs is higher than in autosomes, contrary to findings in humans (Fig. 3C). We investigate the possible causes of this difference in the context of recombination below.

### Mutation rate on the X-chromosome

**Fig. 4:**
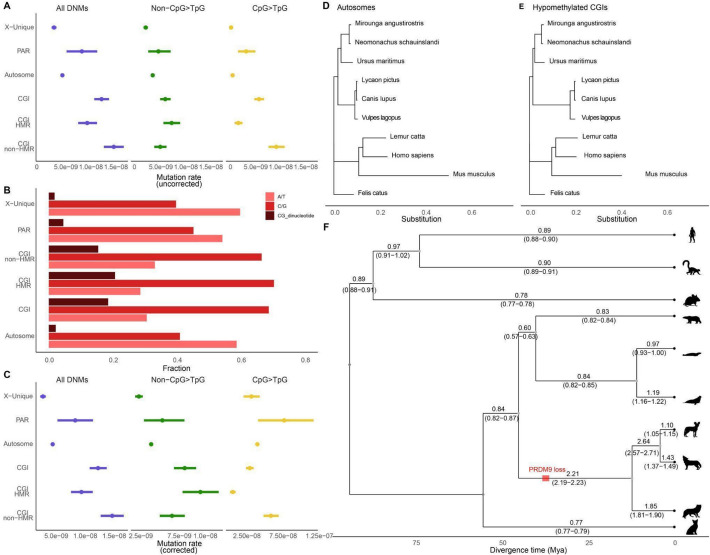
Mutations in different genomic regions. **A** Regional mutation rate (uncorrected for nucleotide composition) of different DNM types. **B** The nucleotide composition of each type of genomic region. **C** Region mutation rates for different kinds of DNMs after accounting for the nucleotide composition in each genome region. **D** Maximum likelihood phylogeny tree built using multiple species alignment of autosomal regions. **E** Maximum likelihood phylogeny tree built using multiple species alignment of hypomethylated CGIs regions in dogs. **F** The ratio between branch length estimated from dog HMR CGIs regions (panel **E**) to branch length estimated from all autosome regions (panel **D**)

Since two copies of the X chromosome occur in females but one copy in males, and given that 71.11% of the DNMs are paternal in origin, the mutation rate on the X chromosome is expected to be lower than that of the autosomes. Following our estimated male-to-female mutation rate ratio of 3.0 (α), we would expect an X-to-autosome mutation rate ratio of 0.83 (95% CI 0.82–0.84, Bootstrap) ([2(2 + α)]/[3(1 + α)]). However, we observe an estimated mutation rate on the X of 3.22 × 10^−9^ (95% CI 2.84 × 10^−9^–3.58 × 10^−9^, Bootstrap), which is 0.66 of the rate on the autosomes of 4.89 × 10^−9^ (95% CI 4.77 × 10^−9^–5.02 × 10^−9^, Bootstrap). Thus, the mutation process on the X chromosome's non-pseudoautosomal regions (non-PAR) is slower than that of the autosomes (Fig. 4A).

The dog PAR is 6.8 Mb [31, 32] on the tip of the X chromosome and has a much higher mutation rate of 8.79 × 10^−9^ (95% CI 6.44 × 10^−9^–1.10 × 10^−8^, Bootstrap), which is 1.8 times higher than the autosomal rate. Since there has to be one recombination event in the PAR in each male meiosis, the PAR should have a recombination rate of approximately 100/6.8 = 14.7 cM/Mb in males and 1 cM/Mb in females, yielding a sex-averaged recombination rate of 7.85 cM/Mb, which is approximately eight times more than the genome average. Given that recombination is mutagenic [6, 33], the high recombination rate in the PAR could explain the higher mutation rate observed in this region.

### Recombination causes increased mutation rate in hypomethylated CpG Islands

Dogs lack *PRDM9*-directed recombination and are expected to have more recombination events in open chromatin, such as in CGIs which are proximal to genes [9, 11]. However, there are more annotated CGIs in dogs than, for example, humans (1.9% of the genome versus 0.8% of the genome), suggesting they may not all be associated with open chromatin and play a regulatory role. We therefore used data from dog sperm sequencing [34] to divide the CGIs into hypomethylated (regulatory, *n* = 30,682,889 bp callable average per individual) and methylated (non-regulatory, *n* = 36,819,203 bp callable average per individual). Figure 4A shows that the elevation in mutation rates in these two classes stems from different factors. In the methylated regions, the higher mutation rate is driven mainly by mutations in a CpG context, which are the product of spontaneous deamination of methylated cytosines [35]. Thus, the methylated CGIs are expected to disappear over time due to the accumulation of these mutations. In contrast, the hypomethylated CGIs show a similarly higher mutation rate in all classes of DNMs. To make a proper comparison, we considered that both the GC content and the proportion of CpGs differ across the genome regions being compared (Fig. 4B). Figure 4C displays the mutation rates after correction, showing a 2.34-fold increase across all mutation types in hypomethylated CGIs. By comparison, non-hypomethylated CGIs exhibit an increase primarily in CpG mutations. We attribute the elevated mutation rate in hypomethylated CGIs entirely to the mutagenic effects of recombination and can derive a rough estimate from this observation. Given that we observe 117 mutations in hypomethylated CGIs, but only expect 50.0 (117*(1/2.34*)) with the genome-wide autosomal mutation rate, this represents a highly significant enrichment (Poisson test, *p* = 2.2 × 10^−16^). There are an excess of 67.0 mutations, or an average of 0.172 mutations per trio, that can be ascribed to the effect of recombination in hypomethylated CGIs. Assuming that all recombination events in dogs are caused by programmed double-strand breaks (both crossovers and non-crossovers) in hypomethylated CGIs, we estimate the mutagenic effect of recombination at 0.8% of all mutations. This value is similar to that recently estimated in humans [36]; however, it is likely underestimated, as some double-strand breaks may also occur in non-hypomethylated CGIs and other regions of open chromatin.

Unlike most mutations accumulated in the germline of males and females from conception to reproduction, since recombination occurs once per meiosis, we expect its mutational contribution to be largely independent of parental age. Therefore, we expect mutations in the PAR and CGIs to be less dependent on parental age than what is observed in the rest of the genome. Moreover, we expect a lower paternal age effect on mutations accumulated in the X chromosome, since the maternal mutation rate has a larger influence on this chromosome. The correlations shown in Additional file 1: Fig. S7 are consistent with these assumptions.

### Estimated age of loss of *PRDM9*

Given the increased mutation rate in hypomethylated CGIs we observe in our dog trios, we would expect a faster relative rate of evolution in the hypomethylated CGIs of species that lack a functional *PRDM9* gene. We sought to investigate this effect by estimating the ratio of CGIs to autosomal substitution rate along the branches of the mammalian phylogeny, including diverse canid species known to lack a functional *PRDM9*. Figure 4D and E depict phylogenetic trees for autosomal and dog hypomethylated regions, respectively, while Fig. 4F shows the ratio of their evolutionary rates. As expected, this ratio is at least twofold higher in the branches connecting canid species that lack a functional *PRDM9* gene, whereas it remains close to one in the branches leading to species with a functional *PRDM9* gene. Notably, we estimate a rate of 2.21 (95% CI 2.18–2.23) in the ancestral canid branch dating back to its split with *Ursus.* The loss of the *PRDM9* gene occurred on this branch, and the elevated rate suggests that the loss of *PRDM9* likely occurred soon after the split (see Methods). These estimates place the loss of *PRDM9* in canids at approximately 37.3 (95% CI 36.3–38.3) million years ago, making it an old evolutionary event.

## Discussion

Our study of mutation rates in 43 dog breeds shows that the mutation rates remain remarkably stable despite the strong artificial selection driven by dog breeding. The only life-history trait associated with mutation accumulation is breed size: Both small and large breeds share the same paternal mutation rate at puberty age, but the smaller breeds exhibit larger age-related slopes in accumulation after puberty. When modeling mutations by parental age, the intercept at puberty age reflects age-independent mutations that are either acquired early in development or from a constant, age-independent influx during gametogenesis, which are indifferentiable in the model. While the paternal mutation rate predicted at puberty does not differ between small and large breeds, and the age of puberty is older in larger breeds than small, the early paternal mutation rate before the age of puberty is hard to compare due to the knowledge gap on how early mutation accumulates. Nevertheless, our results may point to breed-specific growth trajectories. Larger breeds undergo more rapid early cell divisions [37], reach puberty later [38, 39], and have shorter lifespans [39, 40], consistent with the inverse relationship between body size and lifespan [39]. Whether accelerated early growth causally contributes to reduced lifespan remains an important question for future research.

Male dogs accumulate about 1.5 times more mutations in sperm within the testis per year after puberty compared to humans. The higher yearly mutation rate in dogs compared to humans is, therefore, not only an effect of much shorter generation intervals but also of a higher intrinsic mutation rate in dog spermatogenesis, which is conserved across many different breeds. Interestingly, dogs have also been reported to have a higher yearly somatic mutation rate than humans in intestinal crypt tissue, which could partly explain the 5–7 times shorter lifespan observed in dogs compared to humans, despite having similar rates of cell divisions per year [41]. While there is a consistently higher yearly mutation rate in dogs, the difference in mutation per year between dog and human are moderated in the germlines compared to somatic cells. The fraction of mutations acquired early in development appears to be similar between humans and dogs, as there is no difference in the fraction of full-sibling shared mutations. In contrast, the fraction of full-sibling shared mutations appears to be higher in mice and other species with higher fecundity like sticklebacks (12.7–19.5%) [42, 43]and spiders (1–15%) [44]. The shared-mutation fraction estimated in mice and other species is from fewer trios, and the pedigree structures consist of many more siblings; thus, it remains unclear whether the suggested higher early development mutation rate in such organisms is a consequence of biological difference in mutational process or confounded by detection power in sibling-shared mutations. Mutations from gametes of single individuals are needed to quantify early development mutations more strictly than trio-based mutations.

We identified an intriguing case of germline hypermutation reminiscent of recently reported human scenarios [26]. The offspring carried nearly tenfold more de novo mutations than typical, despite no known deleterious environmental exposures, suggesting a genetic contribution. The dam-specific *MLH1 p.D375N* variant driving hypermutation in this first known canine case potentially extends the range of DNA repair genes associated with germline hypermutation and suggests a unique maternal—rather than paternal—mechanism of *MLH1*-mediated germline instability. To our knowledge, this is the first reported association of *MLH1* with germline hypermutation in a dog pedigree, highlighting a new direction for studies of inherited mutation rates across species.

The most conspicuous difference between dogs and humans in the distribution of mutations is the significantly higher rate of mutation observed in dogs in CGIs. We hypothesize that this difference is due to an increased mutation rate caused by programmed double-strand breaks, which in dogs are likely to occur in the open chromatin of hypomethylated CGIs, due to the lack of *PRDM9-*directed recombination. Our estimates suggest this will cause a mutation in the hypomethylated CGIs in one in six births. Since hypomethylated CGIs are functionally important, the added mutation load may help explain why the *PRDM9* system is selectively favored in most vertebrates. We showed that the increased mutation rate in trios is reflected in the accelerated evolution of the hypomethylated CGIs across canid evolution, extending even to their split with *Ursus* about 37 million years ago. Therefore, although the absence of *PRDM9*-directed recombination may be disadvantageous, the canid lineage has managed to thrive without it for a prolonged period, similar to the loss of *PRDM9* in birds more than 65 million years ago.

Our paper had some limitations. For instance, we used the methylation pattern observed in dog sperm as a proxy for the methylation landscape of germline cells. This approach introduces a limitation in the precision of our estimates involving mutation rates in hypomethylated regions of the germline cell, which depends on the degree of concordance between sperm and germline methylation profiles. The observed differences in CpG and non-CpG mutation rates between sperm-based hypomethylated and non-hypomethylated CpG Island regions indicate substantial, but not necessarily complete, overlap between sperm and germline methylation patterns. We consider the estimated timing of *PRDM9* loss to be robust to this approximation, as any systematic bias in methylation patterns is expected to affect estimations in all phylogenetic branches similarly, thereby preserving relative comparisons. However, estimates of the mutagenic effect of recombination are more sensitive to unpairing between the true germline and sperm methylation patterns. Specifically, if the extent of hypomethylated CpG Island regions in germline cells is overestimated based on sperm data, the mutagenic effect may be underestimated—and vice versa. This potential mismatch should be considered when interpreting the magnitude of methylation-associated mutation rate variation.

An additional consideration concerns experiments aimed at identifying crossover recombination events. We pinpointed 7148 crossovers from the autosomes of 390 trios (Methods). We found that three DNMs fell within 10 Kb of the breakpoints. Although this did not show a significant concentration effect, we observe more DNMs than the expected mean of 1.3. Limited by sample size, sequencing quality, and the roughness of the method used to identify crossovers, we were unable to obtain definitive conclusions about the relationship between DNMs and breakpoints as reported in previous human studies [6]. Therefore, we cannot definitively conclude that the elevated mutation rate in CpG regions in the domestic dog results from *PRDM9* gene inactivation. This will be a focus of future research. However, based on the collective observations reported here, such as the faster evolution rate noted in CGIs of species that have lost the *PRDM9* gene, we hypothesize that *PRDM9* plays a role in the unique mutation landscape of dogs.

## Conclusions

In summary, we demonstrate that canine de novo mutations are governed by the combined effects of paternal age-driven replication errors, developmental dynamics linked to body size, and recombination-induced mutagenesis within regulatory CGIs. The absence of *PRDM9* in canids is a natural experiment that reveals the genomic consequences of recombination positioning, with implications for understanding mutation bias in other *PRDM9*-deficient lineages. Our results underscore the stability of mutation rates under artificial selection but also highlight vulnerabilities in functionally critical regions. Future studies will investigate whether analogous mutation patterns emerge in other *PRDM9*-deficient species and assess their broader evolutionary consequences for genome stability.

## Methods

### Sample collection and information

We collected samples from 643 dogs from 43 breeds, including 54 families and 404 trios (Additional file 1: Note S1). Dogs were selected from the Finnish dog biobank, and all dogs in the study originate from Finland. In subsequent analyses, we found that when the sequencing depth for parents in a trio is low, it affects the DNMs calling results. Therefore, we removed 14 trios with low-depth parental samples (average coverage lower than 24 ×). As a result, the final number of trios included in our DNMs analysis is 390 (Additional file 1: Note S1).

### Whole genome sequencing and variant calling of a large cohort of dogs

We used the Covaris system to shear 1–3 μg of DNA into 200–800 bp fragments. The fragments were then sequenced using the Illumina HiSeq 2000 platform with an average depth of 43.3 ×. We subsequently used the bwa mem –M algorithm [45] to map the raw sequence reads to the dog reference genome (Canfam3.1) [46]. We employed PICARD (version 1.96) (https://broadinstitute.github.io/picard/) to remove duplicated reads and merge BAM files for multiple lanes. The sequences were locally realigned and base-recalibrated using the Genome Analysis Tool Kit (GATK, version 3.7–0-gcfedb67) [47]. We recalibrated base quality using GATK BQSR to produce the final BAM files. We then used the HaplotypeCaller algorithm in GATK to perform variant calling and generated a gVCF file for each sample. We genotyped the gVCF files for each trio to generate a raw VCF file jointly. During the base and variant recalibration, we used a list of known SNPs downloaded from the Ensembl database (ftp://ftp.ensembl.org/pub/release-73/variation/vcf/canis_familiaris/) as the training set. Finally, we filtered the raw VCF files based on the following parameters: “QD < 2.0 || FS > 60.0 || MQ < 40.0 || QUAL < 50.0 || SOR > 3.0 || MQRankSum < −12.5” for further analysis.

### De novo mutation calling

We identified DNMs in 404 trios from 54 families using the approach outlined in Additional file 1: Fig S1, adhering to the guidelines and practices from Bergeron et al. [1]. The criteria for DNMs calling using the variant call format (VCF) file of each trio are as follows:

- The offspring genotype is heterozygous (0/1) and the genotype from the same position from both parents is homozygous (0/0).
- The mutation must be supported by a maximum of one read in the parents.
- The genotype quality (GQ) of the DNM is GQ ≥ 40).
- The read depth of any individual in the trio is no less than 12 (min-meanDP = 12), more than half of the average depth of the individual, and not more than twice the average depth (0.5*indDP < DP < 2*indDP)). These depth thresholds are halved for X variants in the chromosomes of male offspring, except for variants in the PAR region.
- The allelic balance, which is the fraction of reads supporting the alternative allele in the child, must be greater than 0.25 and less than 0.75. The allelic balance of variants in the X chromosome of male offspring must be greater than 0.75, except for variants in the PAR region.
- Only single nucleotide mutations are retained.

### De novo mutation filtering

To further remove false positive sites from candidate DNMs, we conducted a filter similar to a manual check with IGV [48]. We used the samtools tool [49] (samtools view) to check the reads of all DNMs. This check is based on the bam file without realignment. We allowed, at most, one incorrect read. Incorrect reads refer to those that differ from the reference allele in the parents and reads that differ from both the reference allele and the DNM in the offspring. Additionally, the offspring’s DNM reads were required to meet the filtering criteria for allele balance, with reads supporting the mutation accounting for 0.25–0.75 of the total number of reads. After excluding unqualified sites, we obtained a final set of 8,565 high-quality DNMs.

### Gene annotation and enrichment analysis

We performed the gene annotation using the Variant Effect Predictor (VEP) [50] in the Ensembl. We use the "maftools" package [51] in R to visualize the results of DNMs annotation and then get a mutational landscape (Additional file 1: Note S2, Additional file 2: Data S2). Enrichment analysis is performed using “g:GOSt” module in g:Profiler [52].

### Germline generationally mutation rates

The mutation rate per base pair per generation was estimated as the number of DNMs divided by twice the number of callable sites. The number of callable sites is the number of sites where we could call a de novo mutation in the whole genome. We calculated the number of callable sites for each trio as positions in the genome where parents are homozygous for the reference allele and that passed the depth filter applied to DNM calling, i.e., no less than 12 and not more than twice the average depth. As in the case of DNM calling, these depth thresholds are halved for X variants in the chromosomes in male offspring, except for variants in the PAR region. We use the term “callable site” to refer to the genome position of a haploid genome that passes our quality filters for de novo mutation calling, and we use the “callable size” to refer to the number used as the denominator in the mutation rate estimate after considering the chromosomal region and individual sex difference. Male individuals possess only one copy of the X chromosome unique region, and female individuals carry two. Thus, the factors for scaling a callable site into a callable size are one and two for males and females, respectively. For all other regions, the callable size for each individual will be two times the number of callable sites extracted. We used generational mutation rates to calculate yearly mutation rates and referred to previous models [53]. The NJ phylogenetic tree of 43 dog breeds was built by SNPhylo [54].

### Mutational class analysis across species

We discretize the 12 different single nucleotide mutations into six mutational classes (C > A, C > G, C > T, T > A, T > C, and T > G), respectively, differentiating C > T mutations in the CpG context (CpG > TpG) from the rest (C > T). We consider DNMs in CGIs as a separate class. We obtained the annotation of CGIs from UCSC (https://genome.ucsc.edu/), using the genome assemblies of canFam3 and hg38 for dogs and humans, respectively. For comparison, we also used previously published DNMs from mice (760 DNMs from 40 trios) [8] and humans (181,258 DNMs from 2976 trios) [6].

We assessed the difference in the fraction of DNMs for each mutational class between species using Fisher’s exact test (R Package stats version 4.1.1). We constructed a 2 × 2 contingency table by dividing the number of DNMs for each species into two categories: those that do and do not belong to the given mutational class: foreground and background, respectively. The resulting *P*-values were adjusted for multiple testing using Bonferroni correction across the 8 mutation classes (significance threshold *P* < 0.05/8 = 0.00625) (Additional file 2: Data S7).

We also compared the rates of DNMs for each mutational class, controlling for differences in the callable fraction of each trio. We also account for differences in the mutational opportunities in the genome and CGIs by scaling the callable fraction of a given mutational class by the proportion of reference bases, i.e., C, T, and CpG, in a given genomic context. To test the statistical significance in differences between mutation rates for a specific mutation class in the entire genome and inside CGIs, we used a binomial test from scipy [55] (version 1.7.3) and adjusted the *P*-values for multiple testing with Bonferroni correction using multiple tests from statsmodels (https://github.com/statsmodels/statsmodels; version 0.13.2).

We investigated the effect of paternal age on the fraction of mutations assigned to each mutational class with linear regression (R Package stats version 4.1.1). We calculated the fractions of DNMs with all observations for a given paternal age and used the total counts for each age bin as weights in the regression.

### DNM shared by siblings

We analyzed 8565 de novo mutations identified through whole-genome analysis. We cataloged each mutation by its unique chromosomal position and filtered for mutations observed more than once. Subsequently, we examined whether mutations shared between individuals were from siblings or half-siblings based on parental information. Siblings were defined as individuals with the same parents, while half-siblings shared only one parent.

All shared mutations in the dataset occurred between either siblings or half-siblings. We identified 79 unique mutations that are shared: 70 are common between two individuals, while nine are shared among three individuals. Notably, only two mutations were found between half-siblings who shared the same father. There are 34 different parental combinations involved in these sibling-shared mutations.

To estimate the fraction of shared mutations between siblings unbiasedly by pedigree structure, we retrieve the number of unique mutations and shared mutations between each full-sibling pair. A mean fraction of shared mutations between a full-sibling pair is then calculated as the total number of shared mutations divided by the total number of mutations (counted by unique position) across all full-sibling pairs. We further bootstrap 1000 rounds of the full-sibling pair mutations to retrieve a 95% confidence interval for the mean estimation of shared mutation fraction between full-sibling individuals.

### Regional mutation rate and in the dog genome

Combining genome annotation from canFam 3.1 and hypomethylated regions from dog sperm samples [34], we split the dog genome into six categories: all autosomal regions (Autosome), pseudo-autosomal regions (PAR), unique regions of the X chromosome (X-unique), CGIs in autosomes (CGI Autosome), hypomethylated CGIs in autosomes (HMR CGI Autosome) and non-hypomethylated CGIs in autosomes (non-HMR CGI Autosome).

Using bootstrap, we then estimate a mean DNM rate with 95% CI in each genomic region. We resampled the 389 trios with replacements for each genomic region to generate a bootstrapped set. In each round of bootstrapping, we estimate a DNM rate using the total number of DNMs and the total callable size of the sampled trios. We bootstrapped 10,000 rounds for each region, and the 95% CI was retrieved using the 2.5% and 97.5% quantiles from the estimated DNM rates.

We also estimate the DNM rate according to the three mutation types (all mutation types, C > T in CG context, and mutations excluding C > T in CG context) to obtain the mutation rate for each mutation class. The 95% CIs of each mutation class are also retrieved from the 2.5% and 97.5% quantiles from the bootstrapped rates. To account for the nucleotide composition difference across different genomic regions, we calculate the fraction of A/T, C/G, and CG dinucleotide sites in the dog reference genome canFam 3.1 for the six genomic regions we categorized. The fractions are further used to scale the callable size in each bootstrap round to obtain the mutation rate per mutation class with nucleotide composition correction.

### Phylogenetic analysis of *PRDM9* loss in dogs

We used the branch lengths of the phylogenetic trees to represent the evolutionary rate. We use the whole genome sequences, CGIs region sequences, and hypomethylated CGIs region sequences to construct ML phylogenetic trees by PhyML [56] and obtain the lengths of each branch. We received the annotation of CGIs and hypomethylated regions [34] from UCSC (https://genome.ucsc.edu/). We divided the CGIs regions into CGI HMR regions and CGI non-HMR regions based on their overlap with HMR regions. We use the same 10 species described above for the evolutionary rate analysis. The whole genome alignment sequences of the 10 species come from the HAL alignment of 241 species zoonomia [57] Cactus alignment. The CGIs alignment sequences are extracted from the HAL alignment using the maffilter tool [58] with the dog as the reference genome. Subsequently, we obtain the species tree with the divergence time of the 10 species from Timetree [59].

We estimated the timing of *PRDM9* loss by analyzing phylogenetic branch lengths from multiple species alignments. Specifically, we performed 100 bootstrap replicates for both hypomethylated CGI regions (based on dog sperm methylation data) and whole-genome regions. Due to computational limitations, the alignment length for both regions in each bootstrap replicate included only 10% of the total length of hypomethylated CGI regions. As a result, the confidence interval is expected to be broader than if the full alignment were used in each replicate.

Our inference relies on the following assumptions: (1) The elevated branch length ratio observed in the ancestral branch shared by dog and African wild dog reflects the full effect of *PRDM9* loss. (2) The branch length ratio in the ancestral branch of all carnivores (excluding cat) represents the scenario where *PRDM9* was still functional. (3) The intermediate ratio in the ancestral branch leading to dog, African wild dog, and fox represents a transition phase during *PRDM9* loss, which is assumed to be an instant event. Violation of these assumptions would systematically bias the time estimation.

To estimate the timing of *PRDM9* loss, we randomly sampled one phylogeny from the hypomethylated CGI regions and one from the whole-genome regions, then resolved the transition point based on the observed branch length ratios. This process was repeated 1000 times to obtain a distribution of inferred loss times (Additional file 2: Data S9). The 2.5th and 97.5th percentiles of this distribution define the confidence interval for the estimated *PRDM9* loss time.

### Identified recombination crossover

First, we used GATK’s PhaseByTransmission to phase our trio pedigrees. We then identified recombination crossover breakpoints based on changes in the offspring’s haplotypes, requiring at least 500 consecutive SNPs of shared origin to define a valid haplotype block. Next, we took the midpoint of the boundaries of two adjacent haplotype blocks as the recombination crossover breakpoint. Our analysis was restricted to autosomes, and we ultimately obtained 7148 crossovers.

## Supplementary Information

Additional file 1: Fig. S1-S7, Tables S1-S2 and Note S1-S5.

Additional file 2: Data S1-S9.

## Data Availability

Raw sequence data is available from the Genome Sequence Archive (GSA) (https://ngdc.cncb.ac.cn/gsa/) under accessions CRA004356 [60], CRA002653 [60], CRA002915 [61];and CRA001113 [61]; and NCBI (https://www.ncbi.nlm.nih.gov/) under the Bioproject PRJNA1079355 [62]. The mapping results, including pre-realignment BAM files (project CRA002655) [60] and post-realignment BAM files (project CRA002654) [60], are deposited in the GSA. Variant calling results (gVCF files and VCF files) are accessible via the Genome Variation Map (https://ngdc.cncb.ac.cn/gvm/) under accession GVM000097 [60] and GVM001075 [60], and the final de novo mutation (DNM) calls are provided in Additional file 2: Data S2. All computational workflows including those spanning read alignment, variant calling, and DNM detection are publicly available in a GitHub repository (https://github.com/1993zsj123/DNMs) [63], and Zenodo (10.5281/zenodo.17051746) [64]. The source code for the analyses script is publicly available under the MIT license. The mouse DNMs were derived from Supplementary Data 1 of ref. [8], while the human DNMs were derived from Data S5 of ref. [6].
