# Supplementary material for: Determinants of de novo mutations in extended pedigrees of 43 dog breeds
Source: Genome Biol. 2025 Sep 25;26:305. doi: 10.1186/s13059-025-03804-2 (PMC12465969; doi:10.1186/s13059-025-03804-2)

Additional file 1

**Determinants of *de novo* mutations in extended pedigrees of 43 dog breeds**

Shao-Jie Zhang^1,2,11^#, Jilong Ma^3^#, Meritxell Riera^3^#, Søren Besenbacher^3,4,5^, Julia E Niskanen^6,7,8^, Noora Salokorpi^6,7,8^, Sruthi Hundi^6,7,8^, Marjo K Hytönen^6,7,8^, Tong Zhou^1,11^, Gui-Mei Li^1,11^, Elaine A. Ostrander^9^, Mikkel Heide Schierup^3^*, Hannes Lohi^6,7,8^*, Guo-Dong Wang^1,2,11^*

**This PDF file includes:**

Fig. S1 – S7

Tables S1, S2

Note S1-S5

**Other supplementary materials for this manuscript include the following:**

Additional file 2: Data S1 – S9


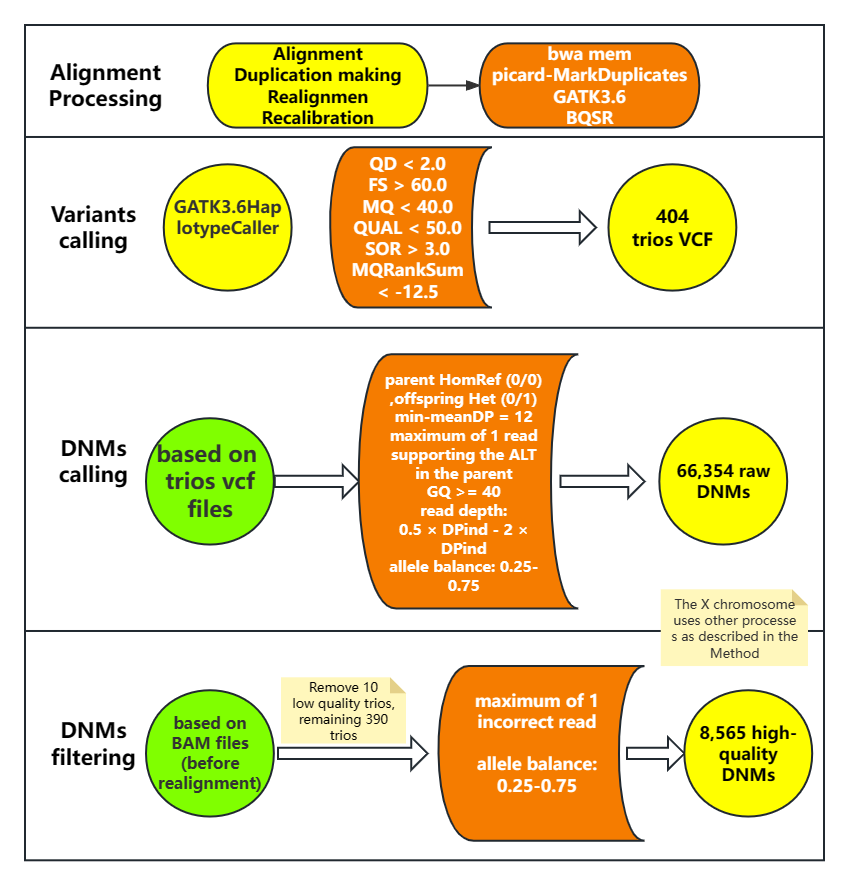


**Fig. S1**

Flowchart for the identification of de novo mutations.


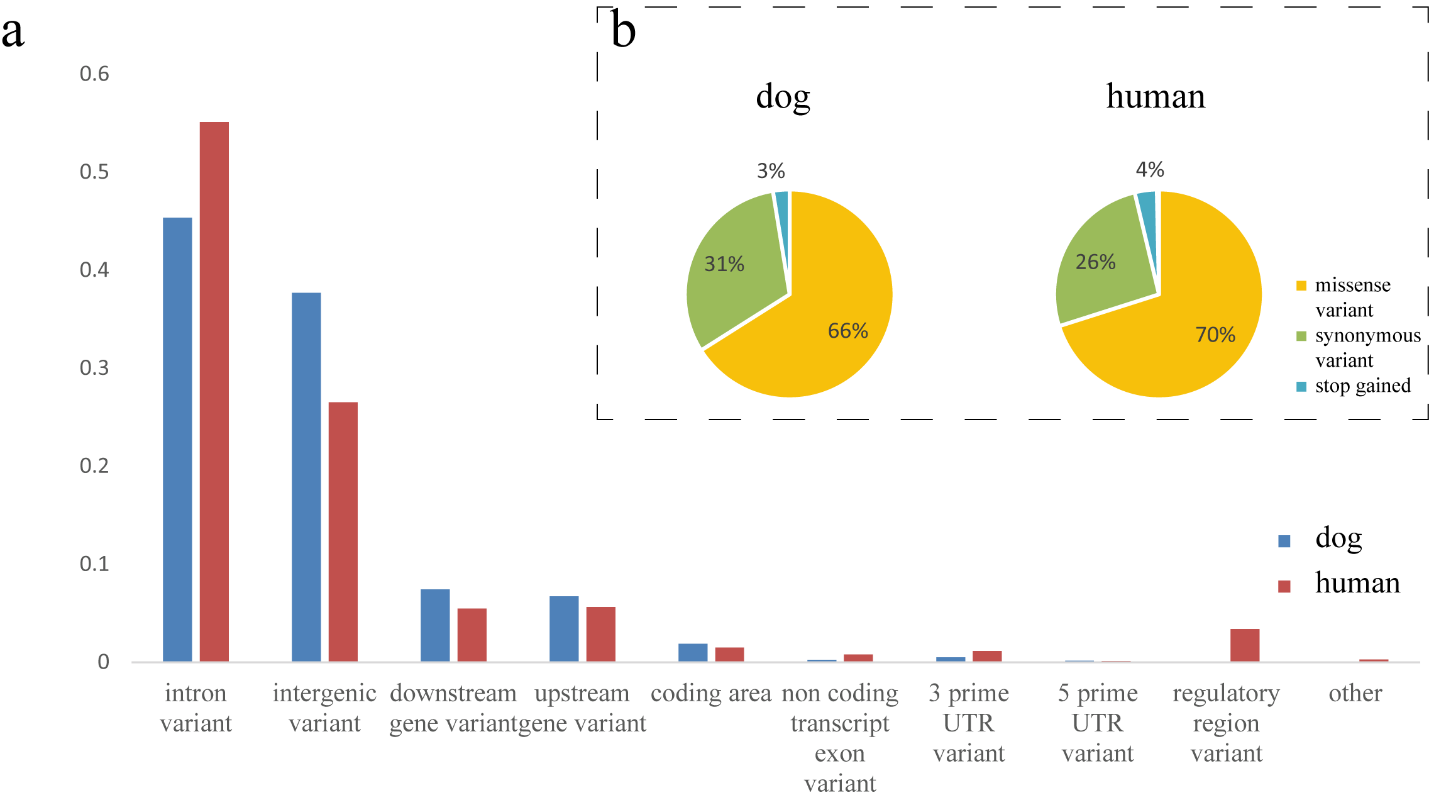


**Fig. S2**

Variant type difference of de novo mutations between dog and human. (a) Comparison in all de novo mutations. (b) Comparison in coding region mutations.


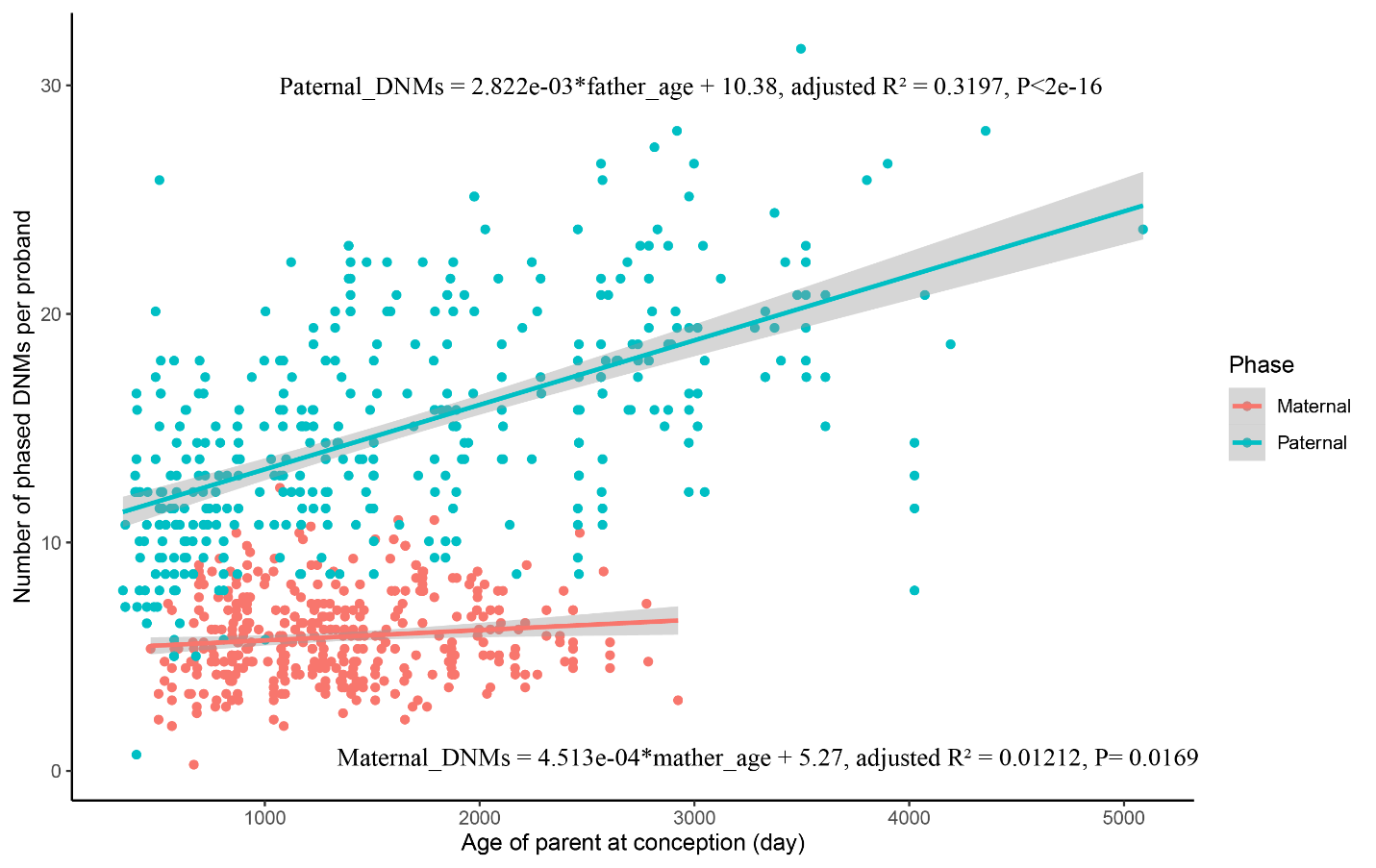


**Fig. S3**

Phased DNMs as a function of the parent’s age at conception.


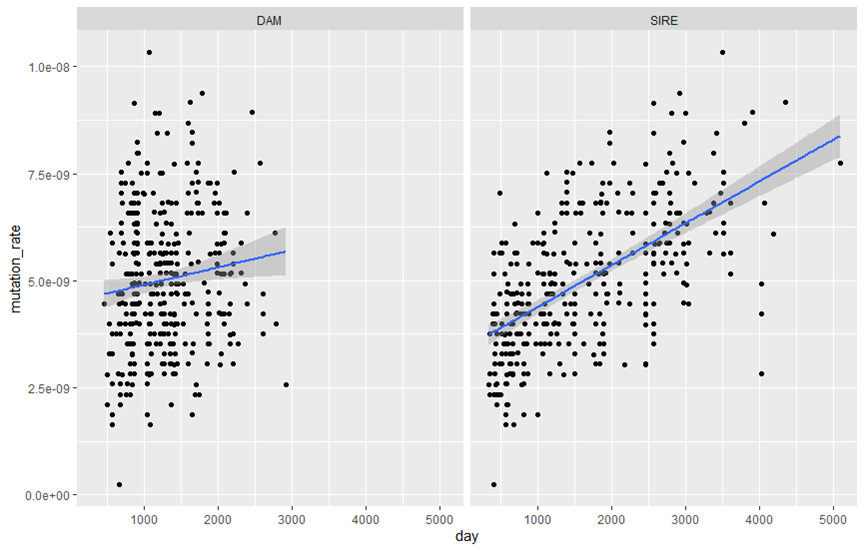


**Fig. S4**

Parent’s age as a function of the mutation rate (per generation).


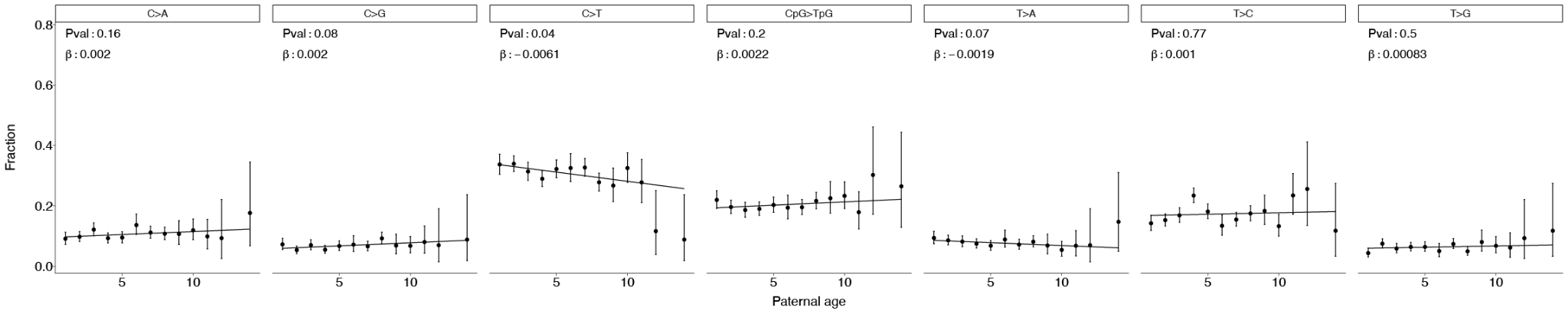


**Fig. S5**

Linear regression on the fraction of DNMs for each mutational class as a function of paternal age, with error bars representing 95% confidence intervals (Binomial).


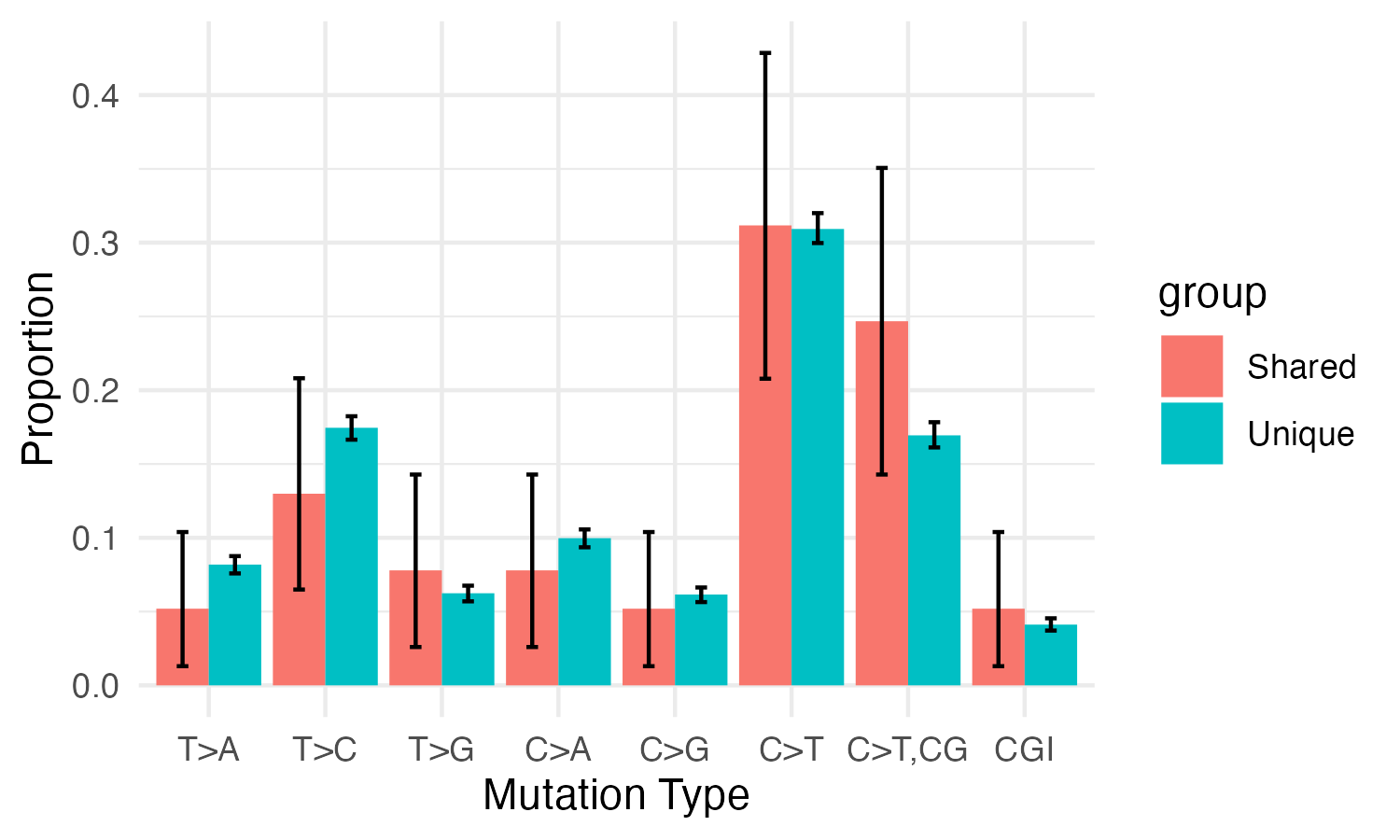


**Fig. S6**

Mutational spectrum in shared mutations and non-shared mutations. CGI for mutations in CGisland regions. The confidence interval is retrieved from 2.5% and 97.5% quantile via bootstrapping 1000 rounds of the mutation in the unique mutation group (8187 DNMs) and shared mutation group (77 DNMs). Details of the bootstrapping results and significance test are provided in Additional file 2: Data S8


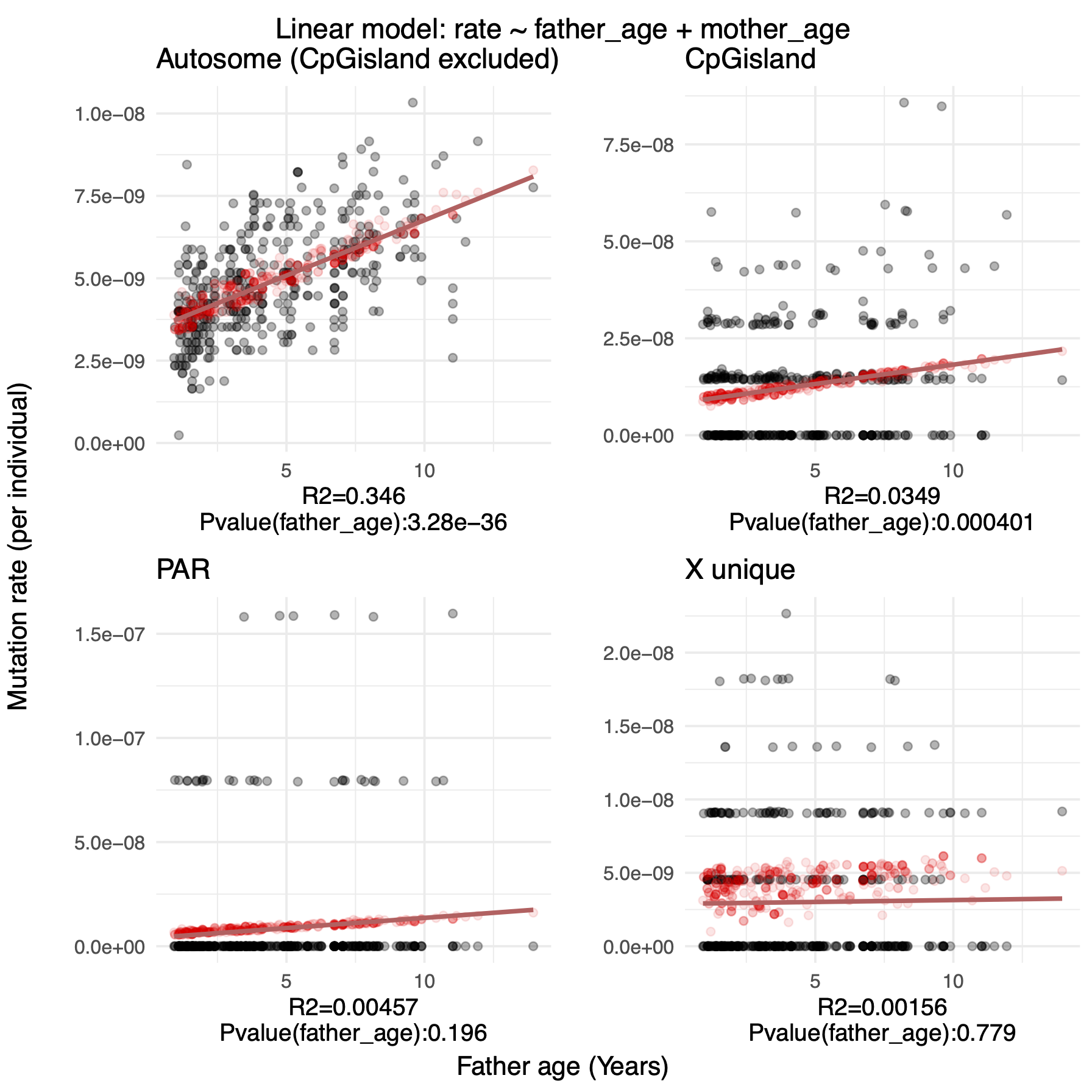


**Fig. S7**

Linear regressions of parental age effect in different genomic regions. The lines note the lineare regression model for paternal age. Each black dot dentes the mutation rate of a trio and each red dot denotes a fitted mutation rate of a trio using the parental age from the linear regression model.

**Table S1**. Recalibration of estimated divergence times in canid.

| Divergence Event | Published Dates (ka) | Novembre's Recalibration (ka) | Recalibration by this study (ka) |
| --- | --- | --- | --- |
| Western Eurasian dogs \| East Asian dogs | LF: 6 (6–11) | 5 (4–17) | 5 (5–9) |
| Mexican wolves \| Yellowstone wolves | BvH: 14 (12–18) | 12 (8–28) | 11 (10–15) |
|  | ZF: 14 (10–17) | 12 (7–26) | 11 (8–14) |
| Basenji \| other dogs | AF: 32 (29–34) | 28 (19–52) | 26 (23–29) |
|  | ZF: 21 (19–23) | 19 (12–35) | 17 (15–19) |
| European wolves \| East Asian wolves | AF: 33 (29–38) | 29 (19–58) | 27 (23–32) |
|  | BvH: 27 (24–30) | 24 (16–46) | 22 (19–25) |
| Dogs \| wolves | AF: 37 (35–40) | 33 (23–62) | 30 (28–34) |
|  | BvH: 28 (24–30) | 25 (16–46) | 23 (19–25) |
|  | ZF: 29 (24–30) | 26 (16–46) | 24 (19–25) |
|  | LF: 34 (17–48) | 30 (11–74) | 28 (14–40) |
| North American wolves \| Eurasian wolves | BvH: 31 (28–32) | 28 (18–49) | 25 (22–27) |
|  | ZF: 31 (29–33) | 28 (19–51) | 25 (23–28) |
| Coyotes \| wolves | BvH: 165 (158–171) | 146 (102–264) | 135 (126–143) |
| Golden Jackals \| Coyote/Wolf ancestors | AF: 995 (797–1,038) | 884 (514–1,596) | 814 (635–870) |

**Table S2.** Predictive accuracy comparison.

|  | **rank** | **elpd_diff** | **dse** |
| --- | --- | --- | --- |
| **Model size** | 0 | 0.0 | 0.0 |
| **Model joint** | 1 | 5.10 | 7.50 |

**rank:** the ranking of the models starting from 0 (best model) to the number of model
**elpd_diff:** the relative difference between the value of LOO for the top-ranked model and the value of LOO for each model.
**dse:** the standard errors of the difference between two values of LOO.

**Note S1: Sample collection**

The dogs were selected from the Finnish dog biobank, which contains nearly 80,000 canine DNA samples donated by private dog owners. All dogs in the study originate from Finland. With an in-house python3 tool, we assembled trios and additional siblings for sequencing from two different types of pedigree cohorts: a pedigree cohort with 3-5 generations, including both parents and littermates and a sire cohort, in which we had sires that had up to 5 litters with different dam over ~10 years period (Fig. 1, Note Fig. 1.1, Additional file 2 : Data S1). We maximized the number of different breeds with various characteristics, such as size and appearance, for the study from the samples available in the biobank. EDTA blood samples were stored at -20°C until genomic DNA was extracted using a semi-automated Chemagen extraction robot (PerkinElmer Chemagen Technologie GmbH). DNA concentration was determined either with NanoDrop ND-1000 UV/Vis Spectrophotometer, Qubit 3.0 Fluorometer (Thermo Fisher Scientific Inc.), or DeNovix DS-11 Spectrophotometer (DeNovix Inc., Wilmington, Delaware, USA).


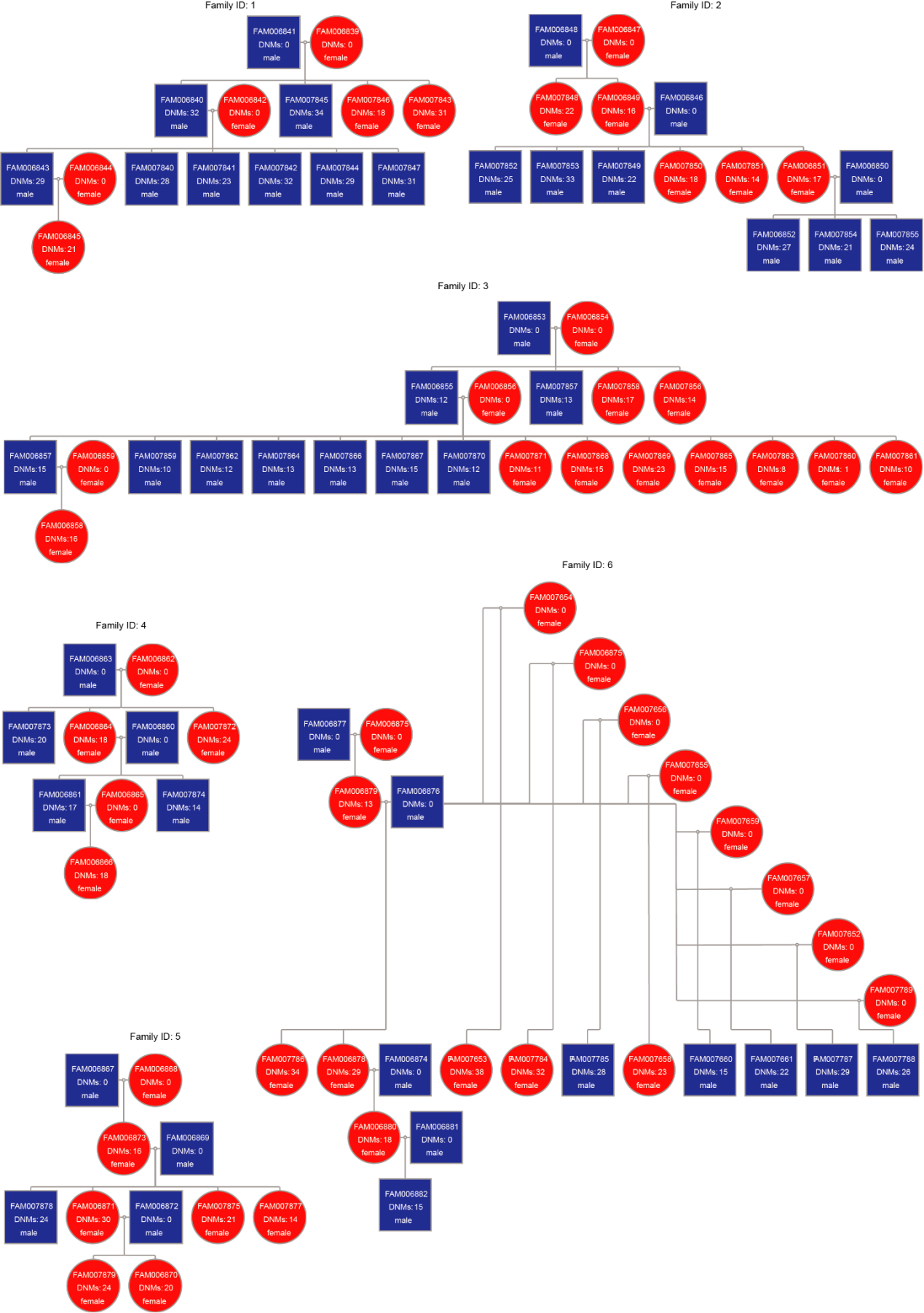


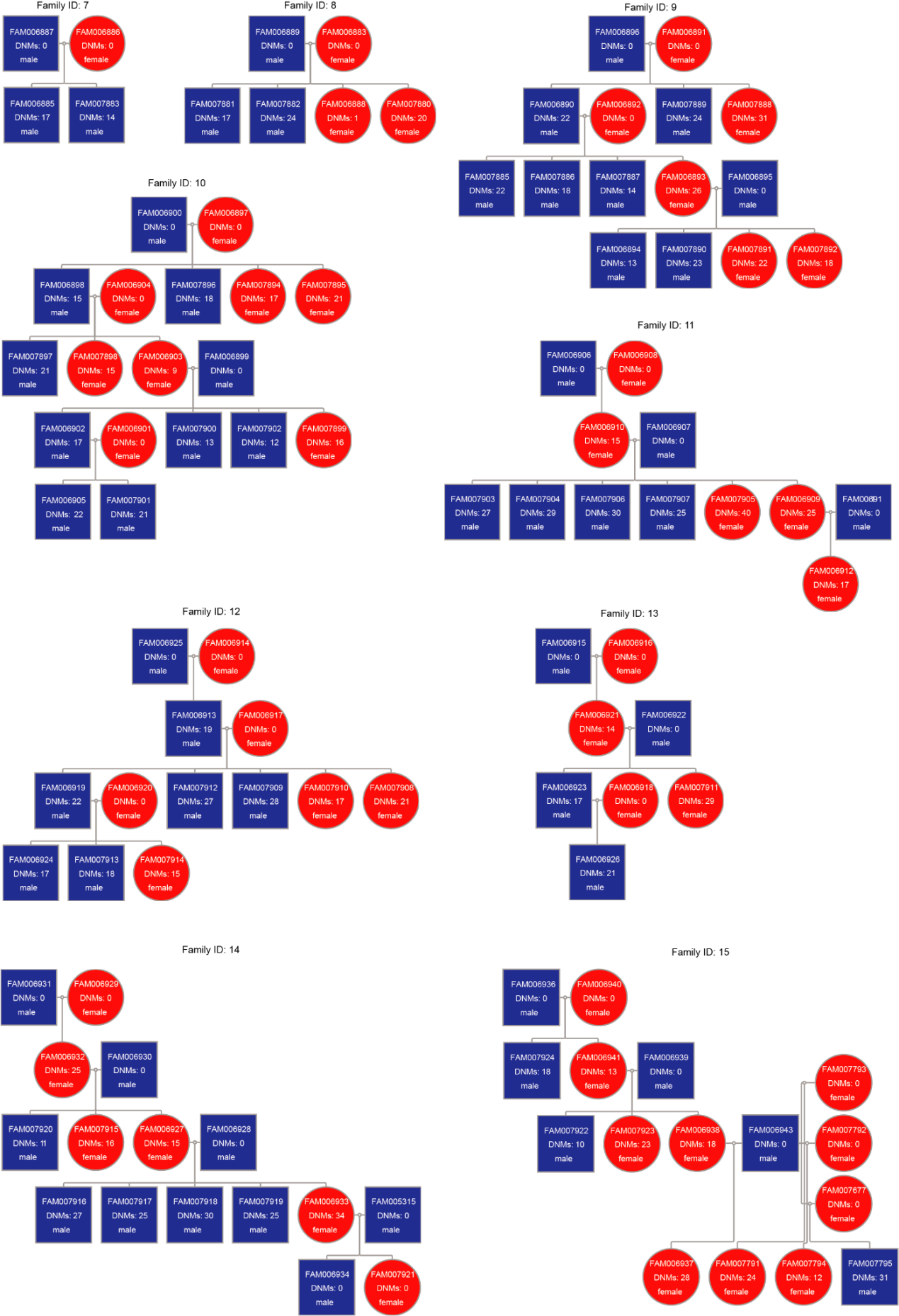


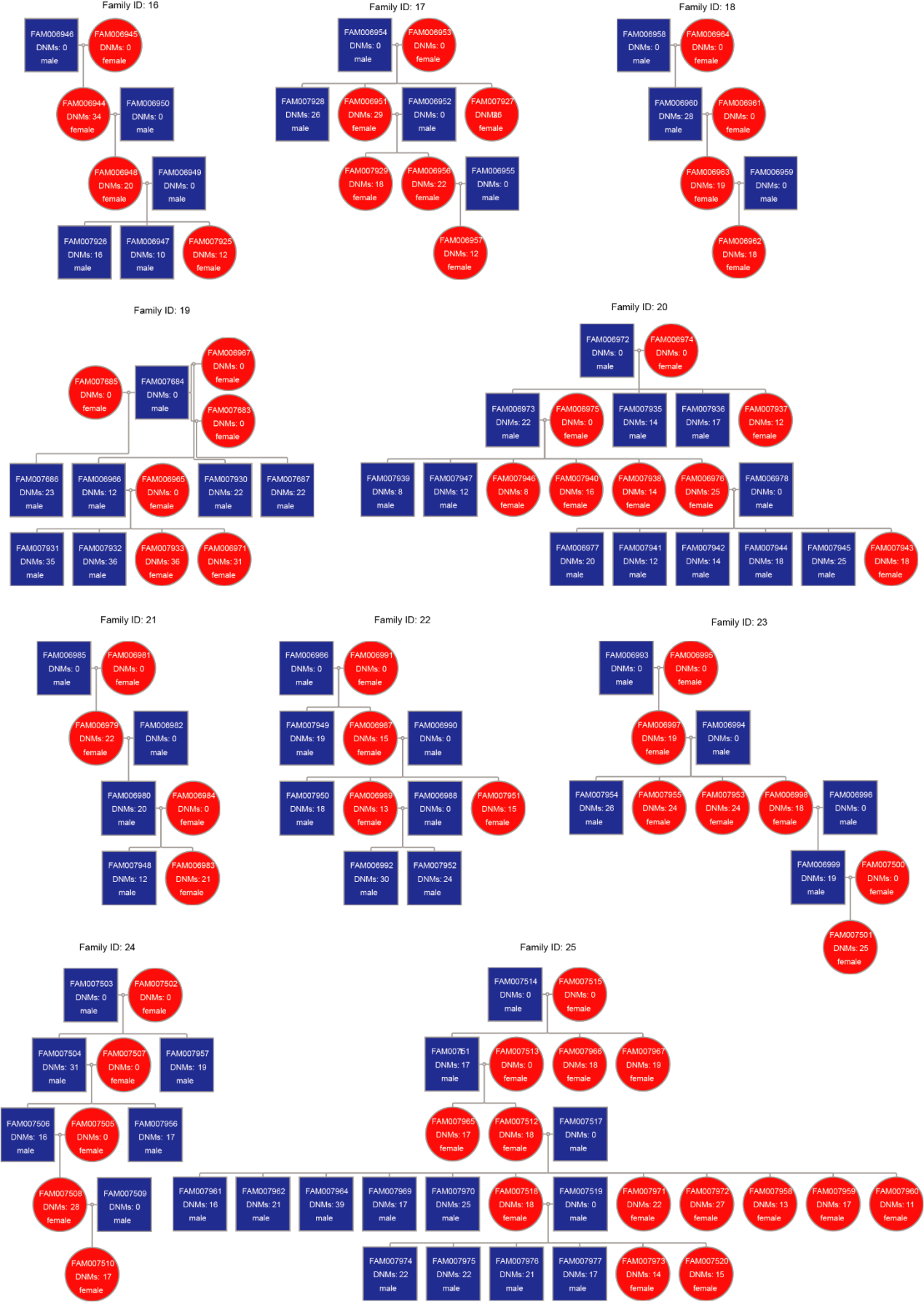


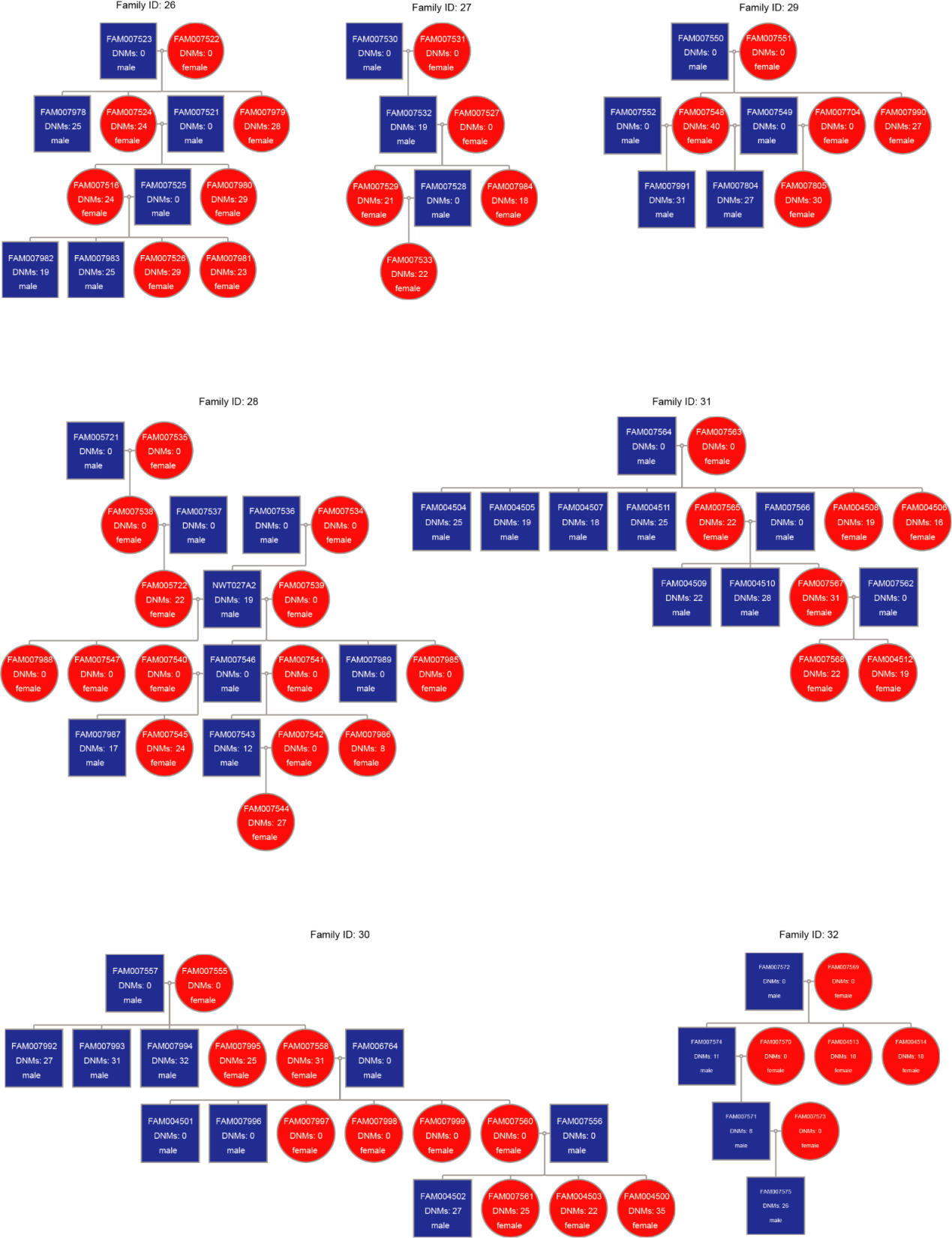


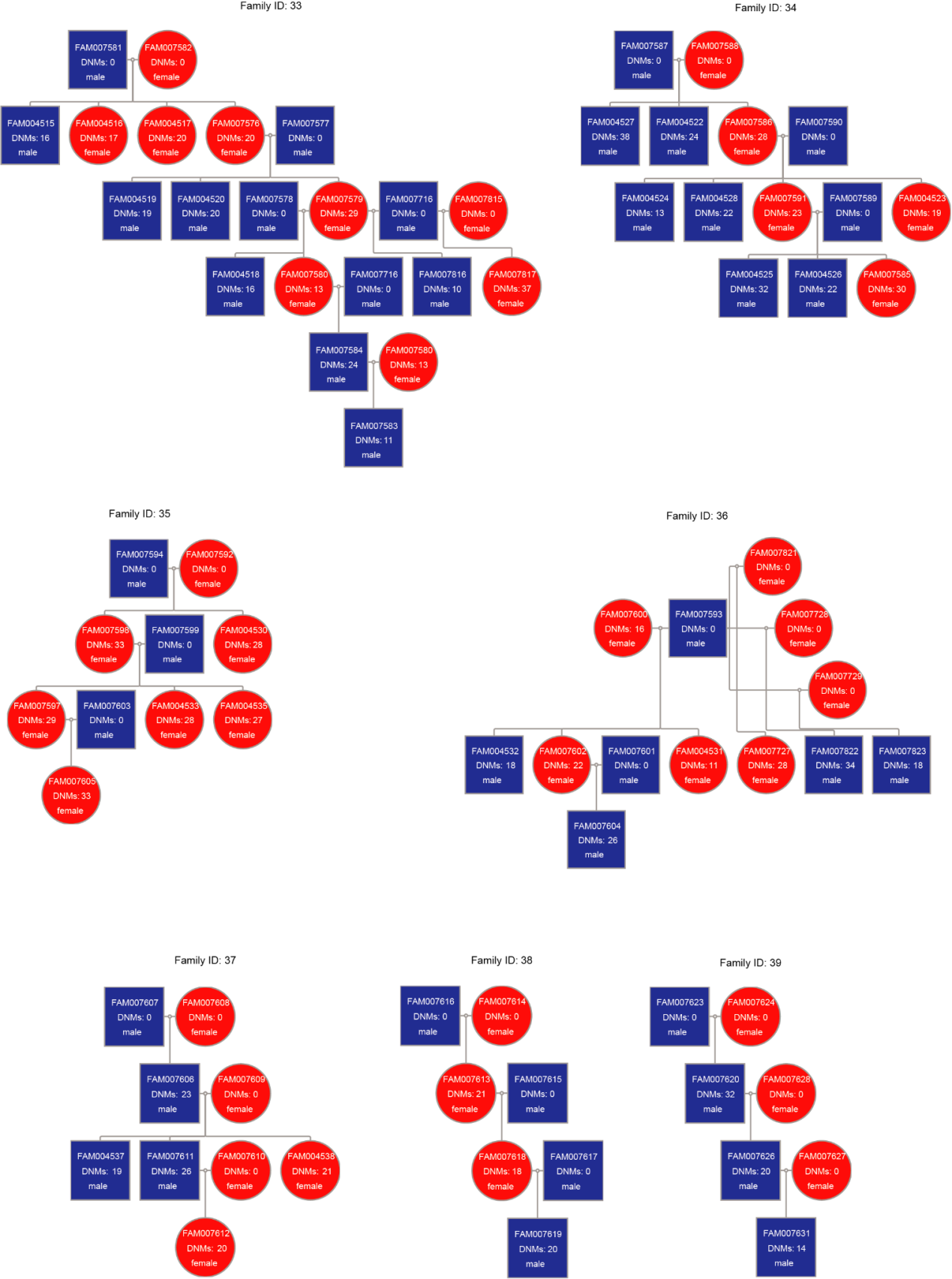


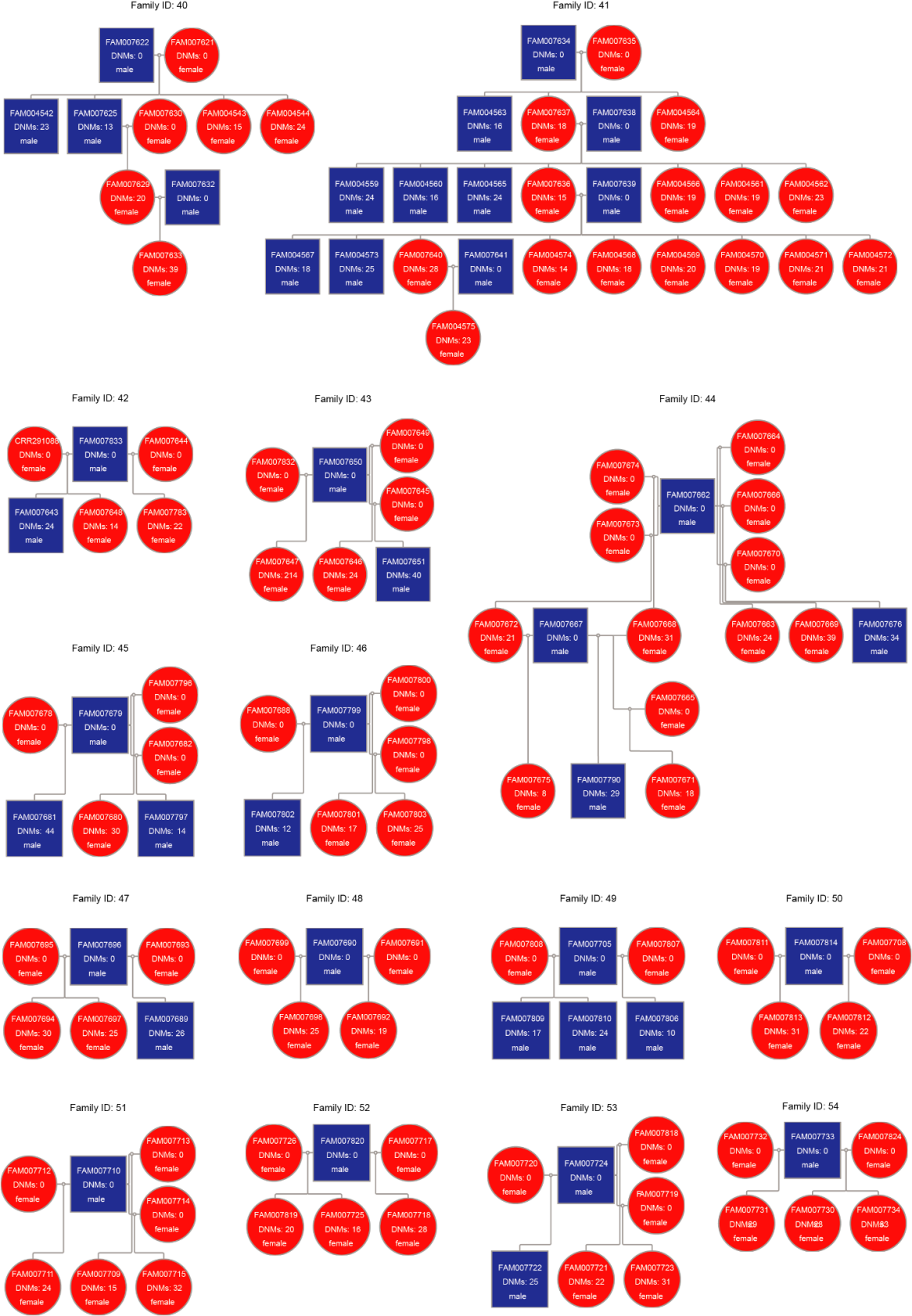


**Note Fig. 1.1.** Pedigrees of 54 domestic dog families.

**Note S2: Mutational landscape and enrichment of hypermutation genes**

We performed the gene annotation using the Variant Effect Predictor (VEP) [51] in the Ensembl. We use the "maftools" package [52] in R to visualize the results of DNMs annotation and then get a mutational landscape. 8,312 dog autosomal DNMs have been found in 3,126 genes, but just 54 genes (1.71%) had more than 3 DNMs in their gene region (Note Fig. 2.1, Additional file 2 : Data S2). We called the gene with unusually large numbers of DNMs the Hypermutation gene. We found 106 hypermutation genes in the human DNMs dataset using the same method, which has over 60 DNMs (Note Fig. 2.2, Additional file 2 : Data S3). Enrichment analysis is performed using “g:GOSt” module in g:Profiler [53]. The hypermutated genes of humans and dogs were enriched in terms of synapse and nervous system development (Additional file 2 : Data S4 and Data S5). Many of these genes play critical roles in neurons and synapses.


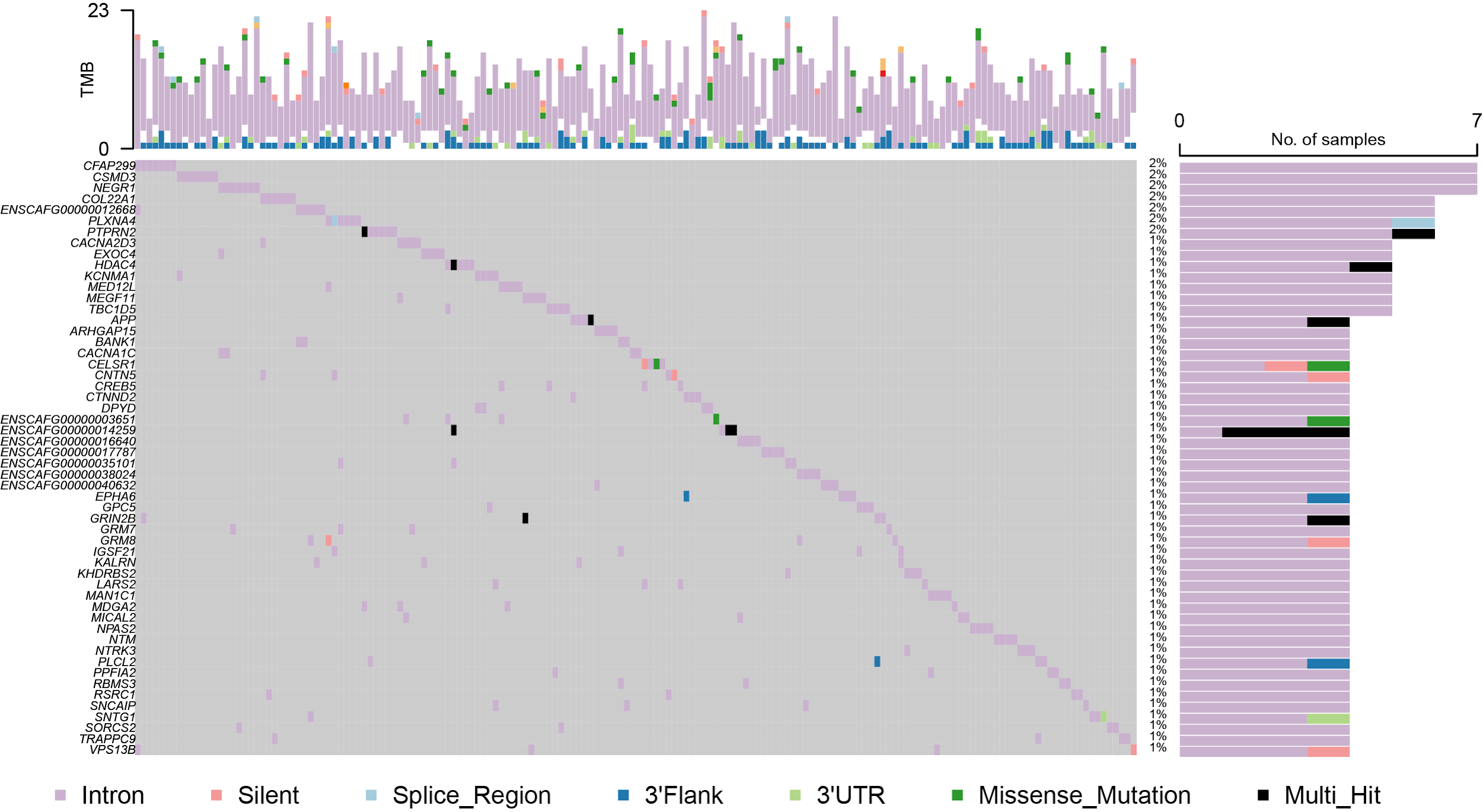


**Note Fig. 2.1.**

Mutational landscape of DNMs (except for the DNMs in intergenic regions). Rows are genes, columns are samples, the right panel shows several samples, and the upper panel shows the number of DNMs in each sample. Different colors represent different mutation types.


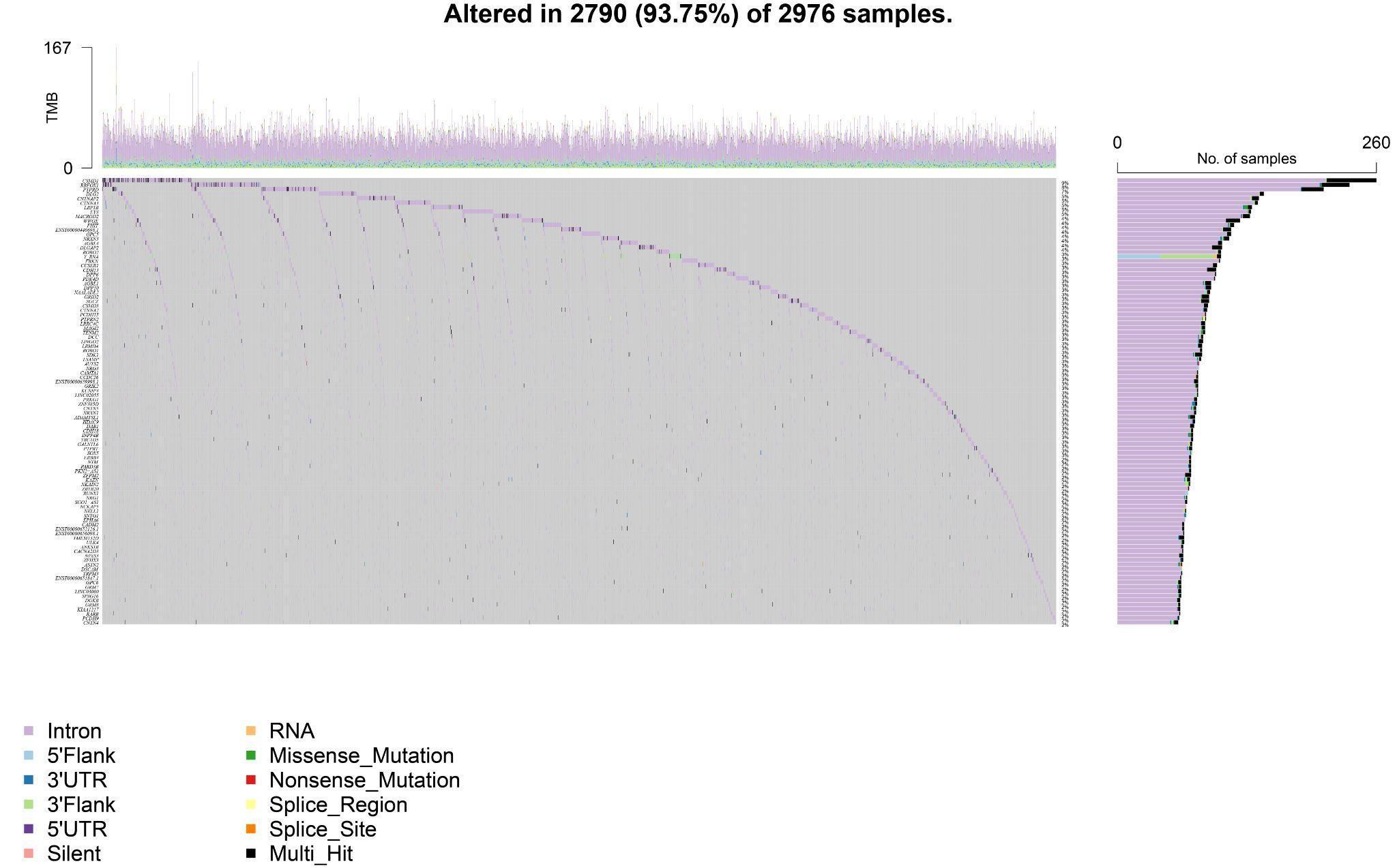


**Note Fig. 2.2.**

Mutational landscape of human DNMs.

**Note S3: Correlation analysis of mutation rate**

Breed effect on mutation rates

We modeled mutation rates for all trios as a function of breed using linear regression, accounting for paternal and maternal ages, with the lm function in R (Note Fig. 3.1, 3.2). We note that the structure of our dataset can bias the results on the breed effect on mutation rates since trios are organized in the litter, and the number of trios per litter is different for each breed. Since multiple trios from the same breed are expected to have the same mutation rate, we accounted for this possible bias using the average mutation rates across litters instead of trios. We also ran an ANOVA analysis on per trio and litter mutation rates as a function of parental ages and breed with the aov function in R (Fig. 2a).

Phenotype effect on mutation rates

We also collected 21 breeds’ characteristics from the American Kennel Club (AKC) and quantified them as phenotypic values of breeds (Additional file 2 : Data S1). We then modeled per litter mutation rates as a function of these phenotypic traits, accounting for paternal and maternal ages, with the lm function in R. (Note Table 3.1).

Randomly select one individual for each breed as the sample set for phylogenetic analysis. Then, The NJ phylogenetic tree was built by SNPhylo [55].

Germline mutation rate by genome regions

We assign the whole genome into four regions, including autosomal regions, CGIs, PAR, and X chromosome unique regions. The autosomal regions contain all autosomes. The CGIs correspond to CGIs on autosomes based on CanFam3.1 annotation. Position 1:680000 from the X chromosome is retrieved as the PAR in our analysis, and the rest of the X chromosome is noted as the unique part of the X chromosome.

Note Table 3.1. The result of 21 phenotype effects on per litter mutation rates

| variable | p-value |
| --- | --- |
| DROOLING_LEVEL | 0.1323 |
| COAT_GROOMING_FREQUENCY | 0.3892 |
| OPENNESS_TO_STRANGERS | 0.4183 |
| ADAPTABILITY_LEVEL | 0.4622 |
| AFFECTIONATE_WITH_FAMILY | 0.4993 |
| SHEDDING_LEVEL | 0.5159 |
| Litter_size | 0.5617 |
| ENERGY_LEVEL | 0.6077 |
| GOOD_WITH_YOUNG_CHILDREN | 0.6213 |
| TRAINABILITY_LEVEL | 0.6764 |
| PLAYFULNESS_LEVEL | 0.7099 |
| Height_male | 0.7198 |
| WATCHDOG | 0.7282 |
| GOOD_WITH_OTHER_DOGS | 0.7286 |
| Lifespan | 0.7646 |
| MENTAL_STIMULATION_NEEDS | 0.7723 |
| Height_female | 0.7784 |
| Size | 0.7956 |
| BARKING_LEVEL | 0.8351 |
| Weight_male | 0.8445 |
| Weight_female | 0.8682 |


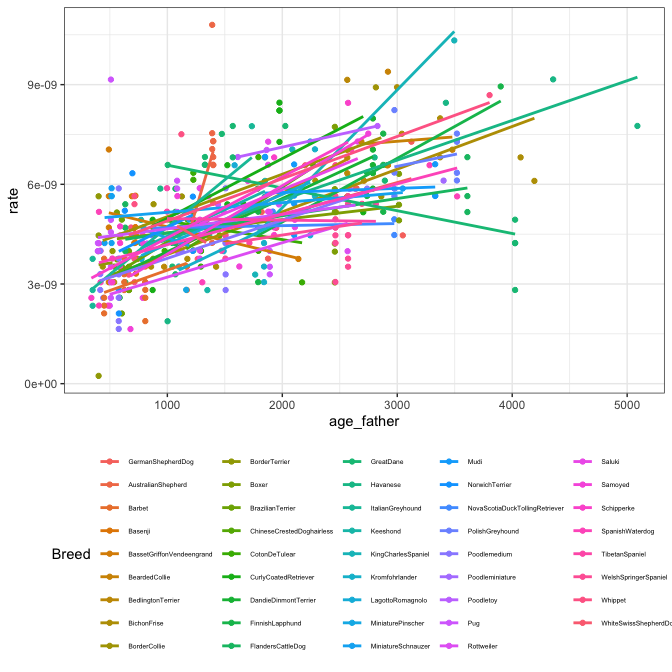


**Note Fig. 3.1.**

Correlation analysis between mutation rate (per trio) and breed.


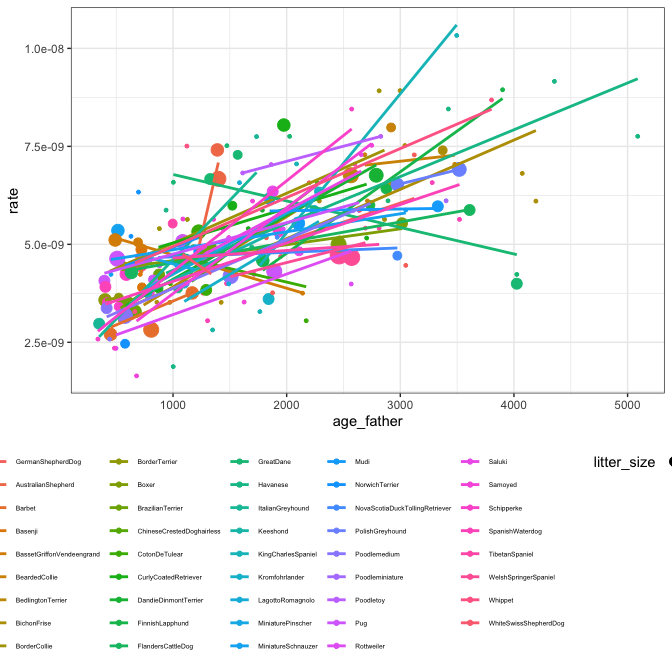


**Note Fig. 3.2.**

Correlation analysis between mutation rate (per litter) and breed.

**Note S4:** **Parental age effect on germline mutation rates**

Linear regression on germline mutation rates

We modeled mutation rates for each region as a function of paternal and maternal ages using linear regression with the lm function in R.

We also modeled mutation rates for each breed as a function of paternal and maternal ages with the lm function in R.

Poisson regression on upscaled DNMs

For this analysis, we only considered trios with phased DNMs, thus retaining 347 dog trios. We modeled the phase-specific accumulation DNMs (
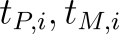
) with Poisson regression. We define lambda (
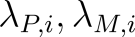
) as a function of paternal and maternal age at conception (
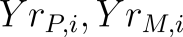
) and account for differences in the callable fraction for each trio.


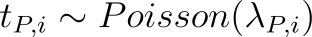

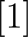


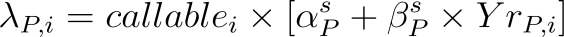

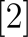


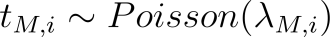

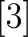


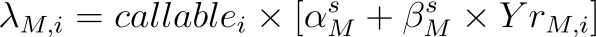

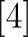


With s being the species in model X, i.e., dog and human, and s being the size group in dogs and species in model X.

For simplification purposes, we scaled
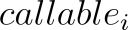
 by 10^-10^, thus moving the range of possible values for
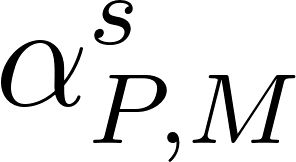
 and
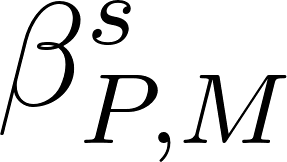
 away from zero. After fitting the model, we scaled the obtained estimates back to the original range.

We define
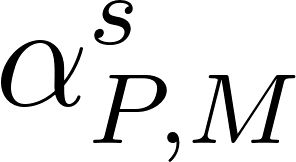
 with a HalfNormal distribution, using a shared hyperprior across species, and
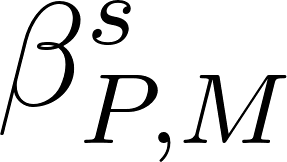
 with an Exponential distribution, parametrized as follows:


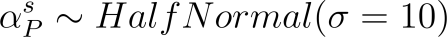

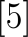


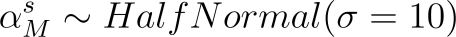

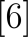


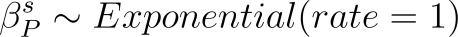

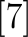


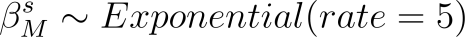

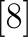


Germline yearly mutation rates.

We calculated germline yearly mutation rates using the predicted mutation rates from our Poisson regression fit model at the average generation time for each sex and species. Following a previous model [54], we accounted for differences in the generation time of males and females ([
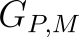
](https://www.codecogs.com/eqnedit.php?latex=G_%7BP%2CM%7D#0)):

[
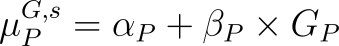
](https://www.codecogs.com/eqnedit.php?latex=%5Cmu_%7BP%7D%5E%7BG%2Cs%7D%20%20%3D%20%5Calpha_%7BP%7D%20%2B%20%5Cbeta_%7BP%7D%20%5Ctimes%20G_%7BP%7D%20#0) [
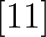
](https://www.codecogs.com/eqnedit.php?latex=%5B11%5D#0)

[
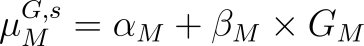
](https://www.codecogs.com/eqnedit.php?latex=%5Cmu_%7BM%7D%5E%7BG%2Cs%7D%20%20%3D%20%5Calpha_%7BM%7D%20%2B%20%5Cbeta_%7BM%7D%20%5Ctimes%20G_%7BM%7D%20#0) [
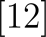
](https://www.codecogs.com/eqnedit.php?latex=%5B12%5D#0)

[
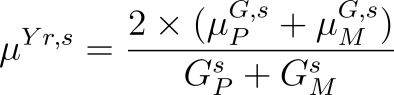
](https://www.codecogs.com/eqnedit.php?latex=%20%5Cmu%5E%7BYr%2Cs%7D%20%3D%20%5Cfrac%7B2%20%5Ctimes%20(%5Cmu%5E%7BG%2Cs%7D_%7BP%7D%20%2B%20%5Cmu%5E%7BG%2Cs%7D_%7BM%7D)%7D%7BG_%7BP%7D%5E%7Bs%7D%20%2B%20G_%7BM%7D%5E%7Bs%7D%20%7D%20#0) [
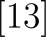
](https://www.codecogs.com/eqnedit.php?latex=%5B13%5D#0)

Downsampling

We did 100 random down-samples of the human DNM dataset to match the number of dog trios with phased DNMs (N = 347) and the average number of DNMs per trio in dogs by dividing the number of DNMs in a given human trio by a factor of 3. For each human down-sample and the dog dataset, we ran a Poisson regression on mutation counts as a function of paternal age using the glm function on R (version 4.2.2). We calculated McFadden’s R^2^ as 1 minus the ratio of the deviance to the null deviance of the model fit (Note Fig. 4.1, 4.2).


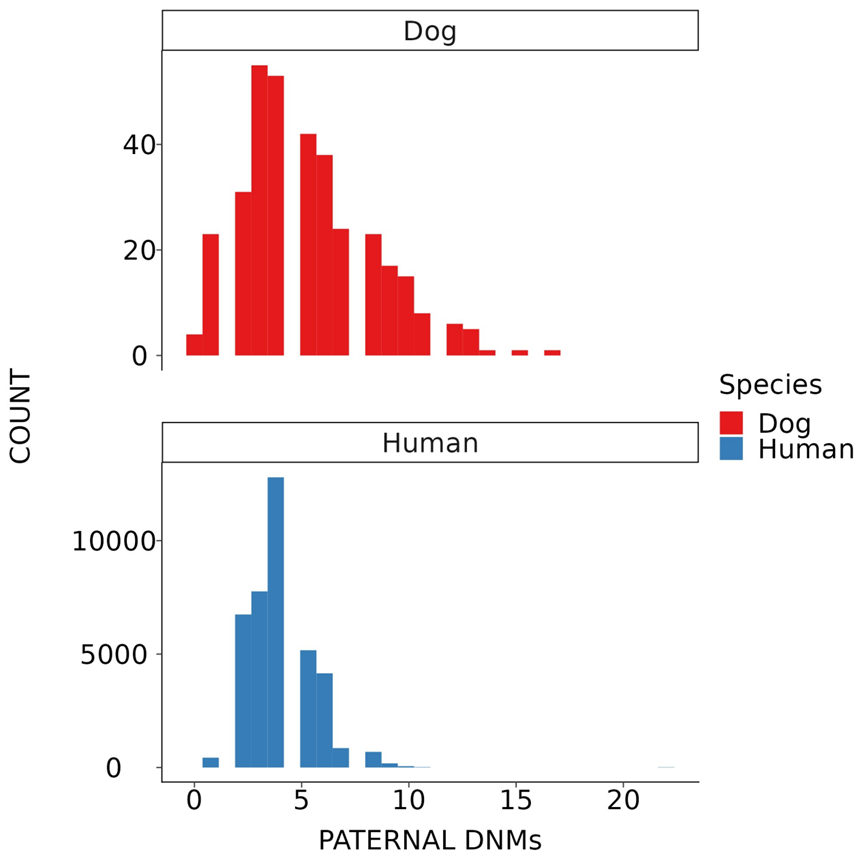


**Note Fig. 4.1.**

Distribution of paternal DNM counts in dogs and humans after down-sampling the human dataset to match the expected number of DNMs found in dogs.


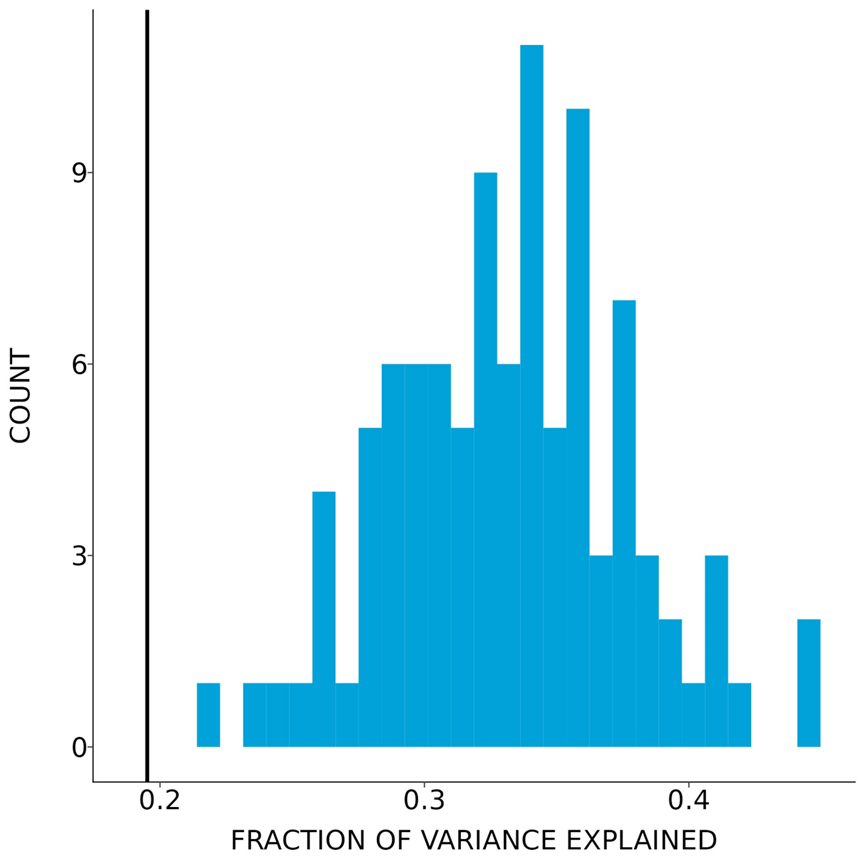


**Note Fig. 4.2.**

The distribution of McFadden’s R2 values in blue obtained from Poisson regression on paternal germline DNMs as a function of the father's age in 100 random down samples of human DNMs to the average number of DNMs in dogs. The value for McFadden’s R2 obtained in dogs is shown in black.

**Note S5 Intercepts, puberty age, and mutation rates**

Comparisons across species and dog breeds are confounded by differences in the age of puberty, which may contribute to the observed positive association between intercept estimates and body size. While large dogs show higher posterior intercept estimates and reach puberty later, this pattern does not extend to medium-sized dogs, whose intercepts are similar to or lower than those of small breeds. Consequently, the association between intercept estimates and age at puberty within dog size categories is not statistically significant (P = 0.25; Fig. 5.1). In contrast, humans have significantly lower intercept estimates despite reaching puberty much later than dogs. When considering both dogs and humans together, we find no significant relationship between intercept estimates and age at puberty (P = 0.26; Fig. 5.2). This suggests a weak positive trend within dogs, but an overall negative trend when comparing dogs and humans.

Because most mutations are age-dependent in both dogs and humans, we observe a strong positive association between the number of paternal mutations predicted at puberty and the age at puberty (P = 6.4 × 10⁻⁵; Fig. 5.3). When examining the per-year accumulation of paternal mutations, we find a significant negative association with age at puberty across humans and dogs (P = 0.04; Fig. 4.4). A similar, though non-significant, trend is observed within dog size categories (P = 0.07; Fig. 5.5). Comparing the per-year mutation accumulation at the time of reproduction to generation time also reveals a significant negative association (P = 0.04; Fig. 5.6), which remains significant when considering differently sized dog breeds alone (P = 0.05; see Fig. 5.7). These findings suggest that dogs accumulate mutations at a higher per-year rate than humans. Within dogs, smaller breeds appear to accumulate mutations more rapidly per year than larger breeds, though this effect is less pronounced in younger individuals, which might reflect a higher mutation rate in an early stage or a higher contribution of age-independent mutations in larger breeds. This pattern is consistent with the significantly higher intercept in larger breeds and the lower paternal age effect estimated in larger dogs compared to smaller ones.

**Note Fig. 5.1.** :


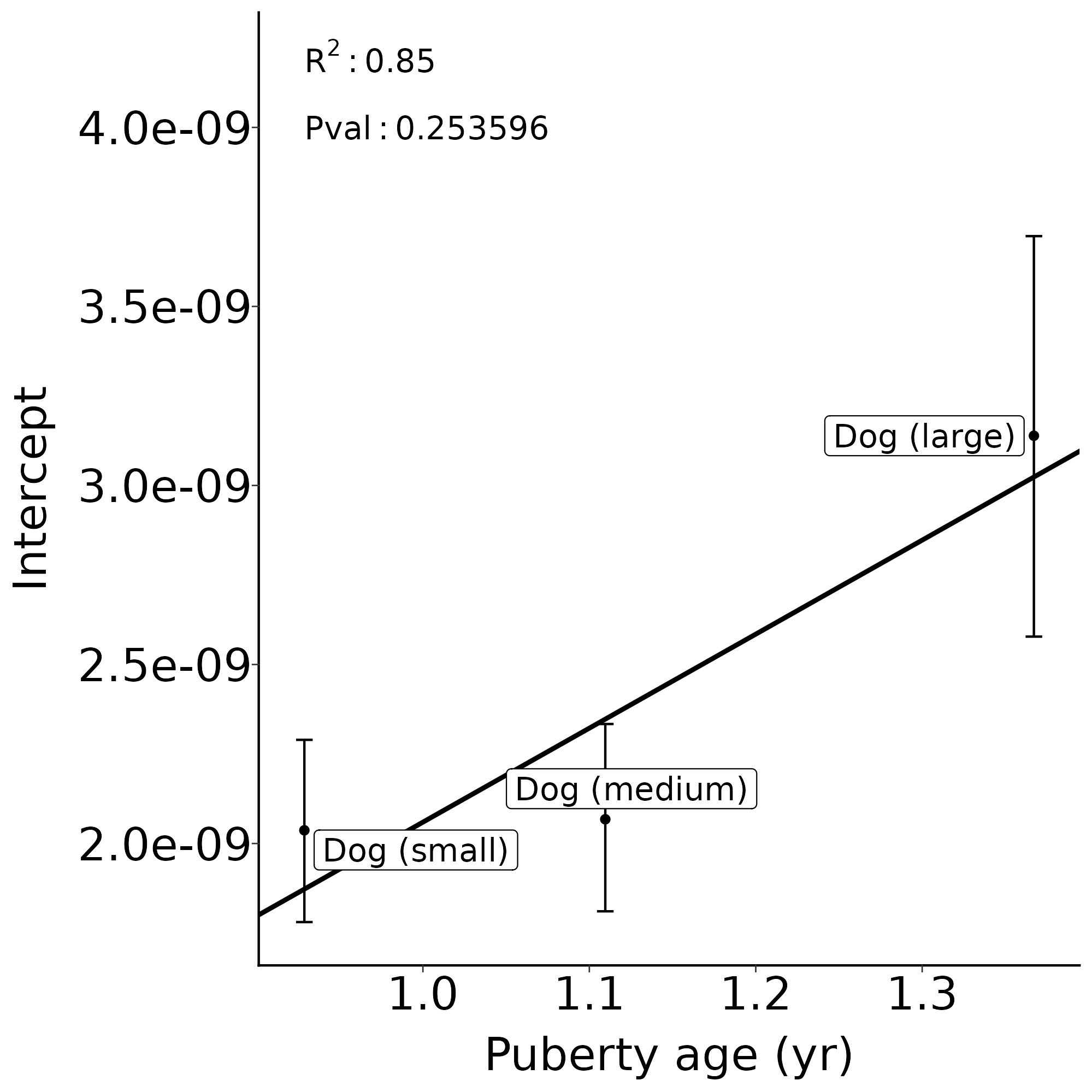


**Note Fig. 5.2.** :


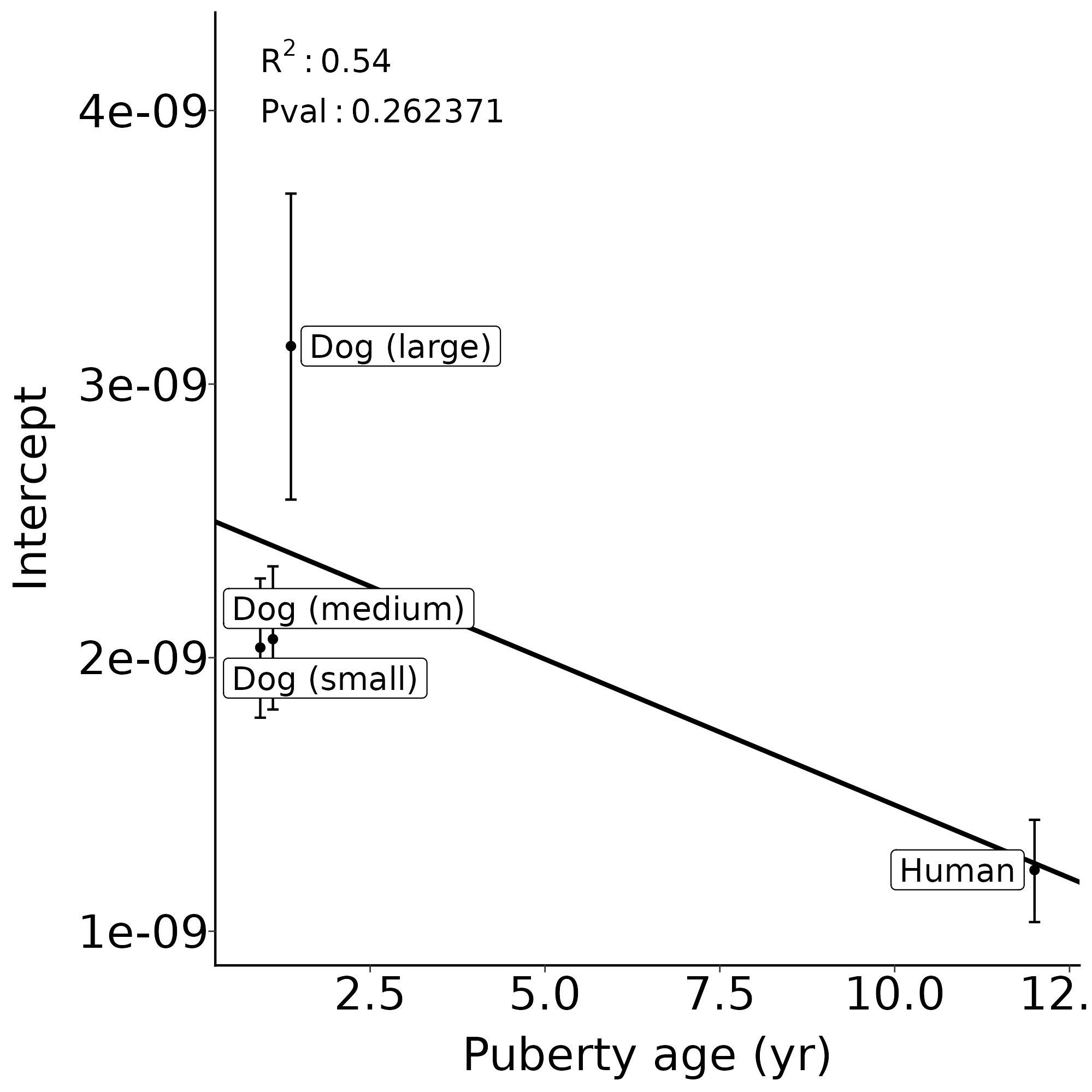


**Note Fig. 5.3.** :


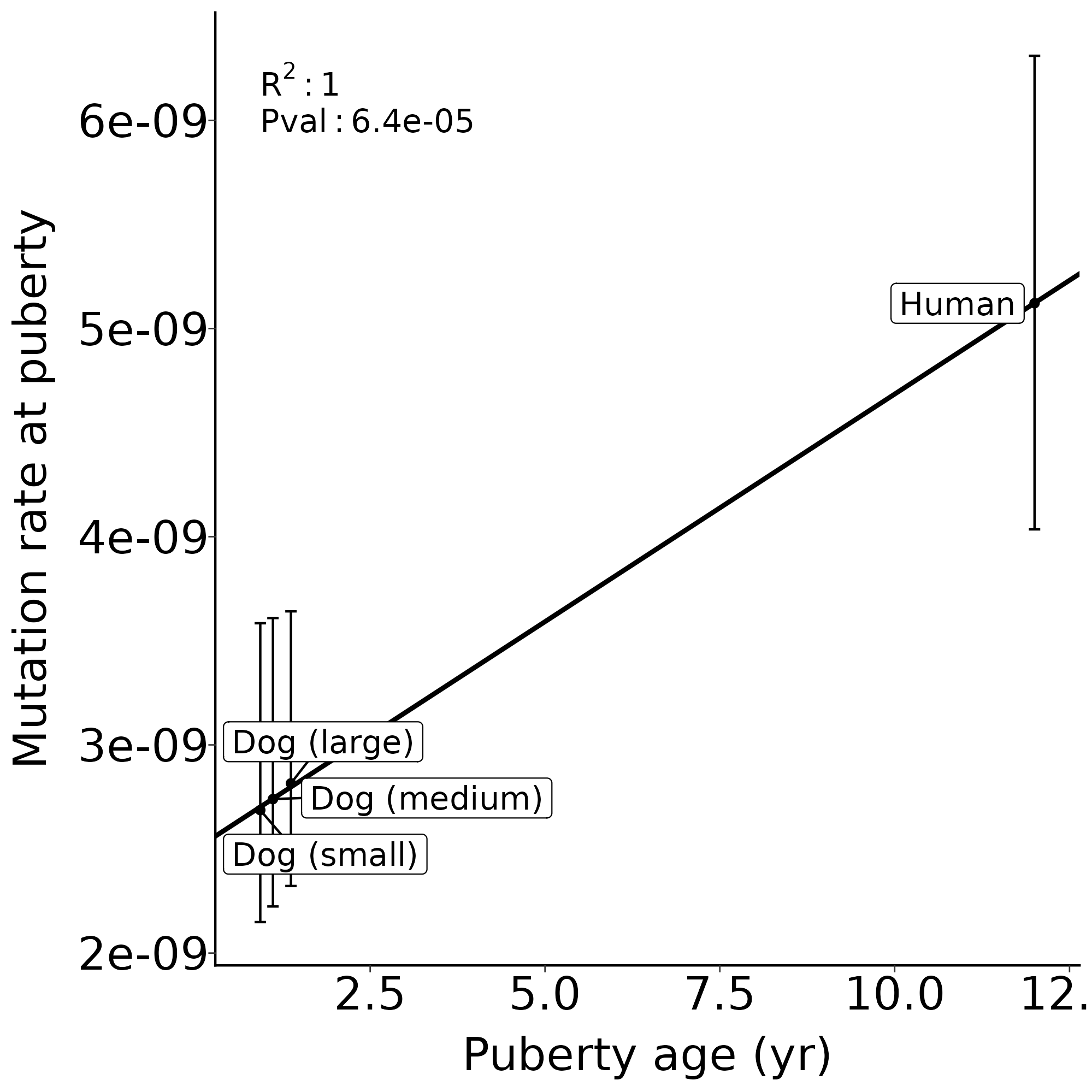


**Note Fig. 5.4.** :


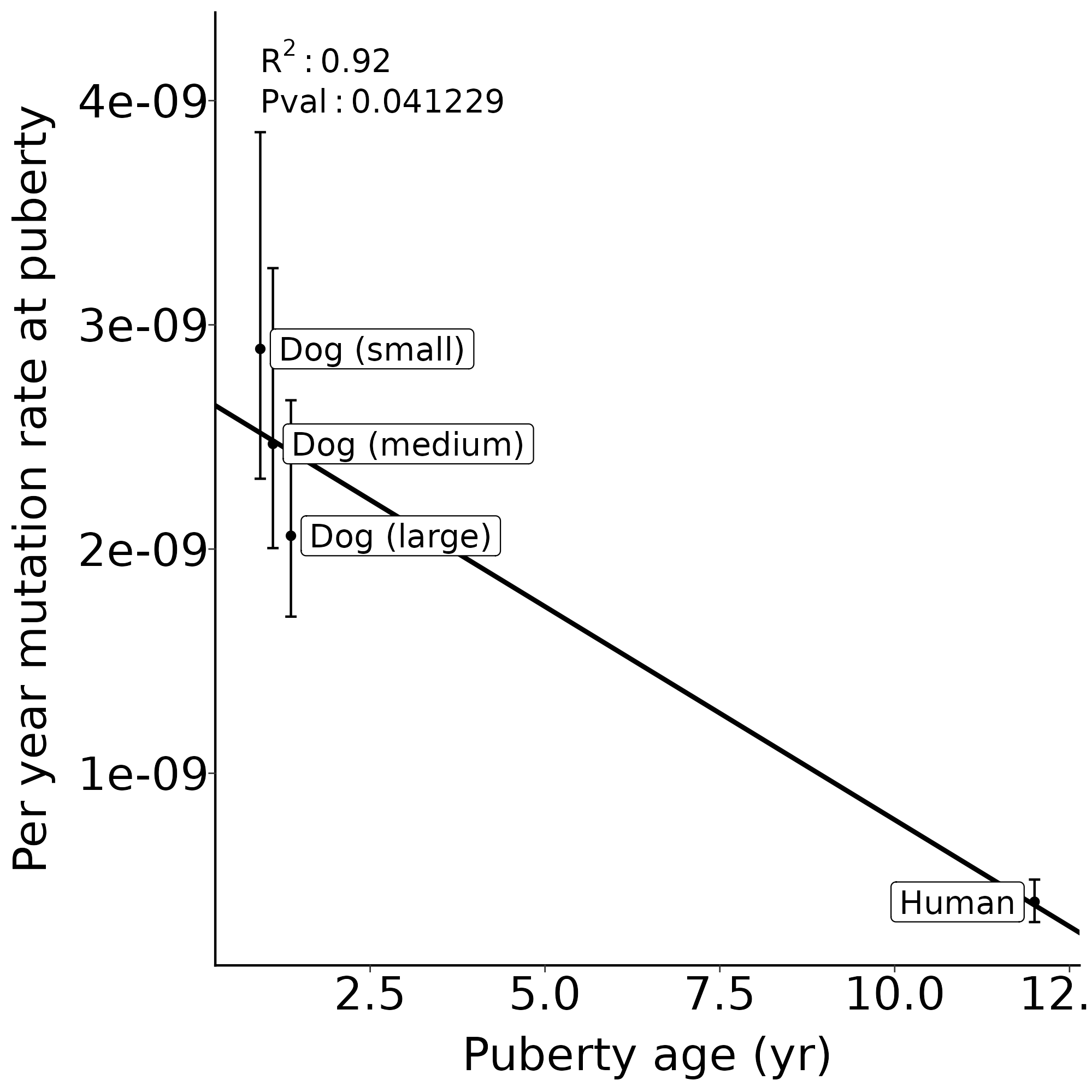


**Note Fig. 5.5.** :


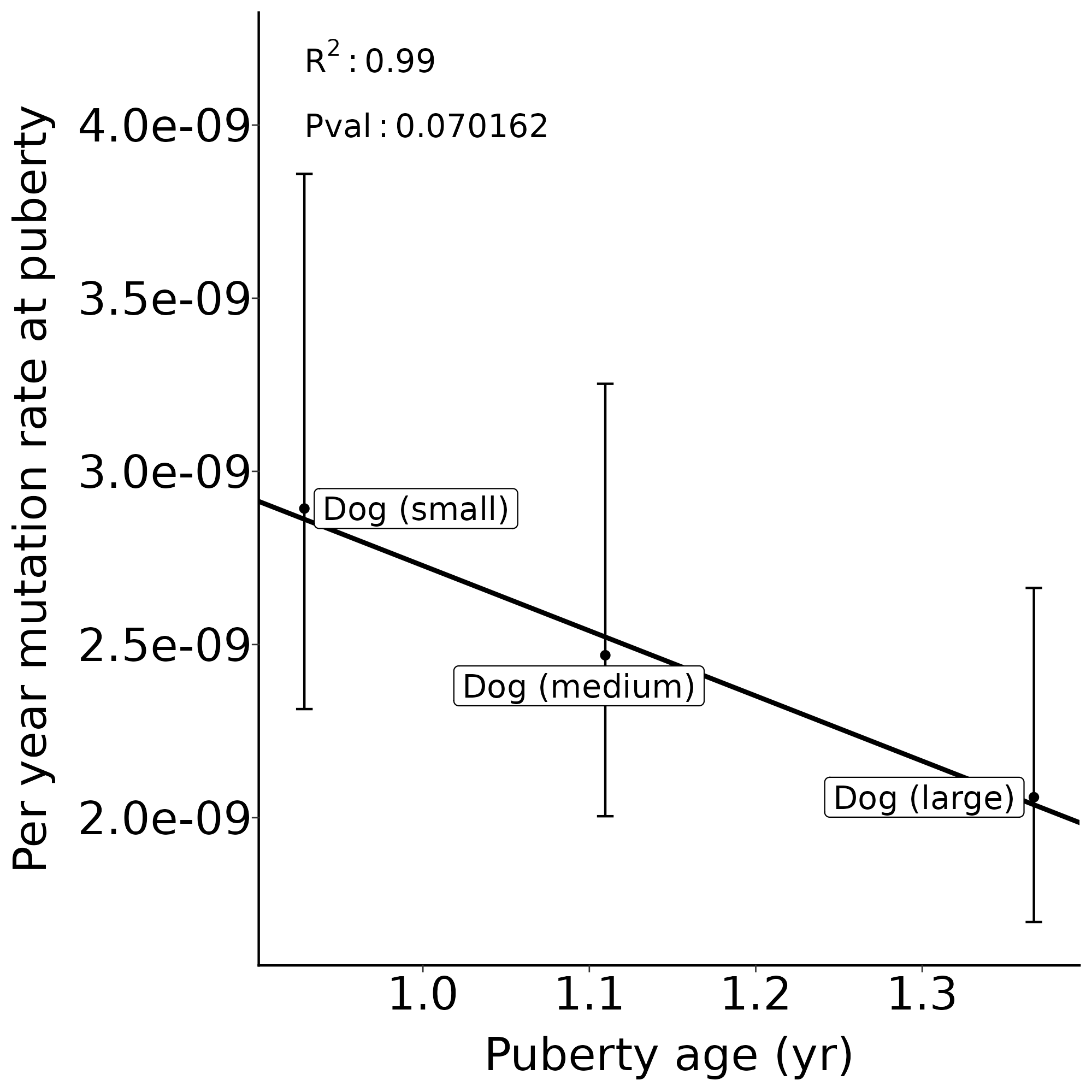


**Note Fig. 5.6.** :


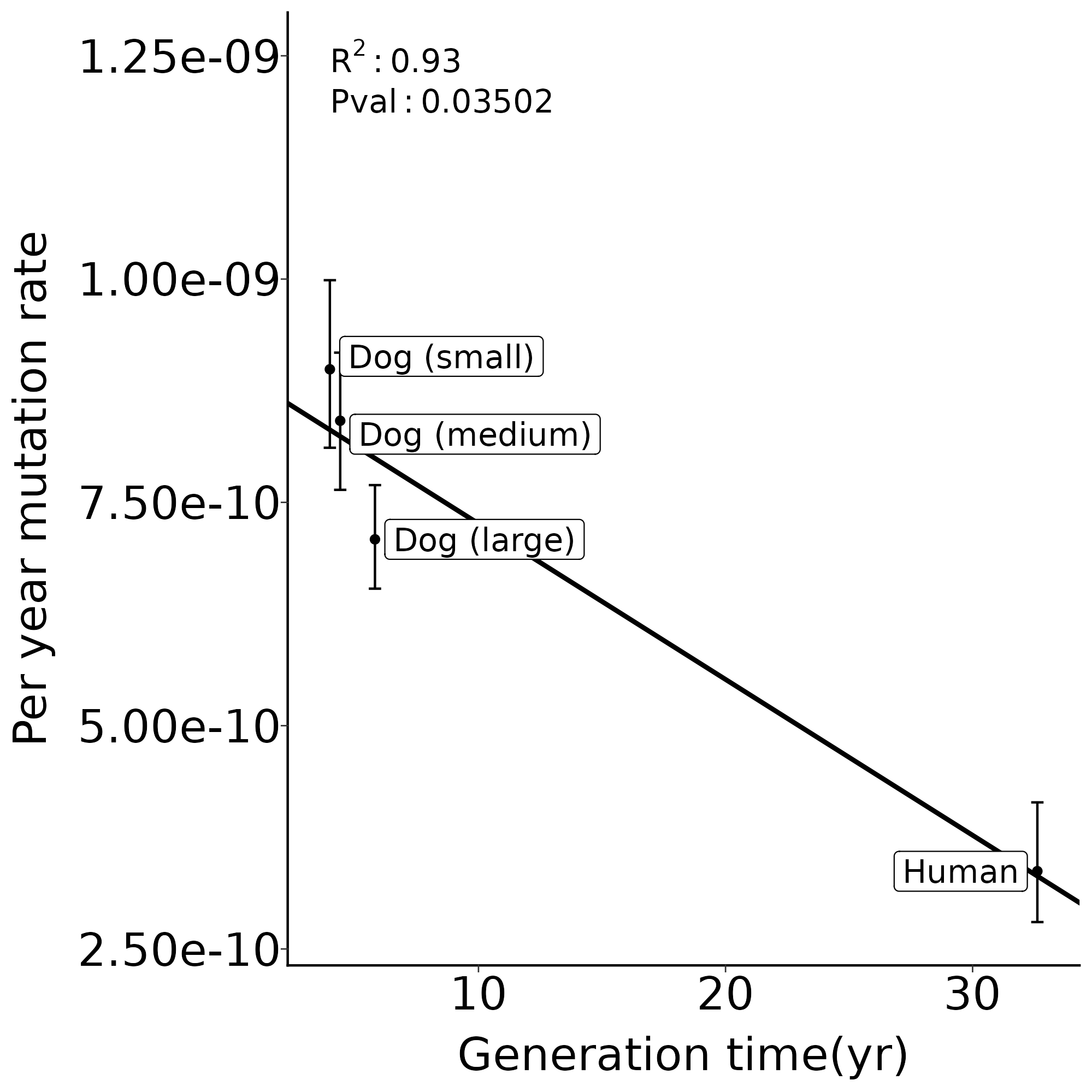


**Note Fig. 5.7.** :


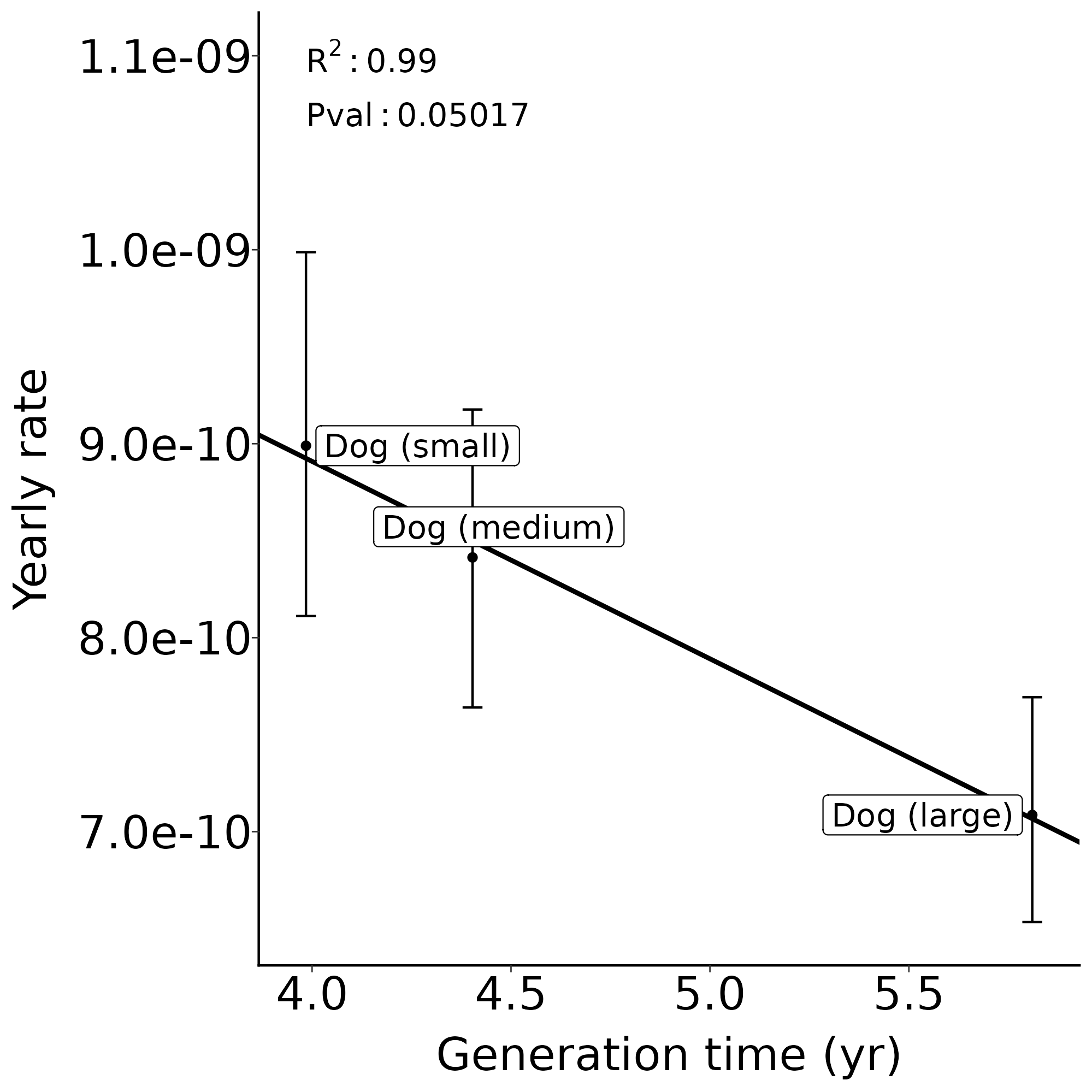

Supplement: Supplementary file 1 — Additional file 1: Fig. S1-S7, Tables S1-S2 and Note S1-S5. [file 13059_2025_3804_MOESM1_ESM.docx]
